# Burden and trends of ovarian and uterine cancer due to high body mass index from 1990 to 2021: an age–period–cohort study based on the GBD 2021, and projections through 2036

**DOI:** 10.3389/fonc.2025.1647757

**Published:** 2025-08-22

**Authors:** Bo Yin, Huijuan Zhou

**Affiliations:** Department of Gynecology, Shanghai Key Laboratory of Maternal Fetal Medicine, Shanghai Institute of Maternal-Fetal Medicine and Gynecologic Oncology, Shanghai First Maternity and Infant Hospital, School of Medicine, Tongji University, Shanghai, China

**Keywords:** high body mass index, ovarian cancer, uterine cancer, Disability-adjusted life years, global burden of disease

## Abstract

**Background:**

Ovarian cancer (OC) and uterine cancer (UC) are significant public health concerns among women of reproductive age. High body mass index (BMI) contributes to the increasing burden of these cancers globally, but comprehensive epidemiological assessments remain limited.

**Methods:**

Data were obtained from the Global Burden of Disease (GBD) Study 2021 (1990–2021). Deaths, disability-adjusted life-years (DALYs), and corresponding age-standardized rates (ASRs) were calculated to assess disease burden. Burden by age was analyzed in five-year intervals. Differences across Sociodemographic Index (SDI) quintiles were compared. Temporal trends from 1990 to 2021 were assessed using estimated annual percentage change and absolute number changes. Future ASRs were projected to 2036 using a Bayesian age–period–cohort model.

**Results:**

In 2021, an estimated 2,022 (95% uncertainty interval [UI], 473–3,611) deaths and 99,915 (95% UI, 22,387–178,579) DALYs from OC attributable to high BMI occurred among women of reproductive age, with Eastern Europe exhibiting the highest age-standardized mortality rate and DALYs among 21 GBD regions. For UC, there were 2,202 (95% UI, 1,545–2,910) deaths and 114,117 (95% UI, 80,122–150,221) DALYs, with the Caribbean among the regions with the highest burden. From 1990 to 2021, ASRs for mortality and DALYs for both OC and UC attributable to high BMI significantly increased and are projected to continue rising over the next 15 years. The burden increased with age, peaking at 45–49 years. Regionally, burden rose in low- and middle-SDI areas, while high-SDI regions showed initial increases followed by declines.

**Conclusions:**

The rising mortality and DALYs of OC and UC attributable to high BMI, especially in low- and middle-SDI regions and high-burden areas such as Eastern Europe and the Caribbean, highlight an urgent need for targeted prevention and weight management interventions among women of reproductive age.

## 1 Introduction

The global epidemic of overweight and obesity has become a pressing public health challenge, with its prevalence nearly tripling since 1975 (1, 2). Among women of reproductive age (15–49 years), high body mass index (BMI) is particularly concerning, as it is closely linked to a range of adverse health outcomes, including cardiovascular diseases, metabolic disorders, infertility, and various cancers (3, 4). Accumulating evidence has demonstrated that high BMI is a significant modifiable risk factor for both ovarian cancer (OC) and uterine cancer (UC), two major gynecological malignancies that collectively contribute to substantial cancer-related morbidity and mortality worldwide (5–7). Recent large-scale cohort studies have further substantiated the association between elevated BMI and increased risk of gynecologic cancers, reinforcing the urgency of addressing this modifiable risk factor in global health strategies (8–13).

Despite growing recognition of the association between BMI and cancer risk, global epidemiological assessments of the burden of OC and UC attributable to high BMI remain limited, especially in terms of long-term trends, age-specific impacts, and regional disparities. Moreover, the burden in low- and middle-SDI (Socio-demographic Index) regions is likely underestimated due to limited cancer surveillance infrastructure (14–16). Quantifying the disease burden in these populations is crucial for identifying vulnerable groups and informing targeted interventions and policy responses.

In this study, we used estimates from the Global Burden of Disease (GBD) Study 2021 to evaluate the global, regional, and national burden of OC and UC attributable to high BMI among women of reproductive age from 1990 to 2021. We examined trends in age-standardized mortality and disability-adjusted life-years (DALYs) across SDI levels and age groups and projected the global burden through 2036 using a Bayesian age–period–cohort model, aiming to provide timely and actionable evidence for cancer prevention and control efforts.

## 2 Methods

### 2.1 Data sources

This study was conducted in accordance with the Strengthening the Reporting of Observational Studies in Epidemiology and Guidelines for Accurate and Transparent Health Estimates Reporting guidelines to ensure transparency and methodological rigor. All analytical procedures were systematically recorded, and the data sources were clearly specified. The analysis draws upon estimates from the Global Burden of Diseases, Injuries, and Risk Factors Study 2021 (GBD 2021), published in 2024 (17). GBD 2021 offers a systematic evaluation of 371 diseases and injuries across 204 countries and territories, covering the period from 1990 to 2021 (18). It includes a wide range of health metrics such as incidence, prevalence, mortality, years lived with disability, years of life lost, and DALYs. The relevant data were retrieved through the Global Health Data Exchange (GHDx) platform (http://ghdx.healthdata.org/gbd-results-tool).

### 2.2 GBD estimation framework

The GBD 2021 study, supported by over 11,500 collaborators from 164 countries, provides a comprehensive and internally consistent assessment of global health loss (19). The estimation process integrates a vast array of data sources, including population-based surveys, hospital and clinical records, civil registration and vital statistics systems, disease surveillance databases, scientific literature, and government reports (https://ghdx.healthdata.org/gbd-2021/sources) (17). To ensure consistency and comparability across regions and time periods, all data inputs are mapped to standardized diagnostic categories using the International Classification of Diseases framework.

The core estimation strategy involves the use of sophisticated statistical modeling tools. For mortality estimates, the Cause of Death Ensemble Model is employed to identify the best-performing models across a range of candidate specifications. For non-fatal outcomes such as incidence and prevalence, the DisMod-MR 2.1 Bayesian meta-regression tool is used to pool data from disparate sources, while accounting for measurement error, sampling variance, and between-study heterogeneity (20).

All GBD estimates are accompanied by 95% uncertainty intervals (UIs), derived from 1,000 draws from the posterior distribution of each model (21). These intervals represent the 2.5th to 97.5th percentile range, capturing the degree of uncertainty around each estimate.

Importantly, the GBD does not provide direct comparisons of disease burden between normal and high BMI groups. Instead, attributable burden is estimated using population attributable fractions based on a theoretical minimum risk exposure level, which for BMI is set at 20–25 kg/m². This means our estimates reflect modeled burdens relative to this optimal BMI range rather than direct subgroup comparisons.

Detailed modeling approaches for OC and UC are documented in the methodological appendices of the GBD 2021 study (https://www.healthdata.org/gbd/methods-appendices-2021/cancers).

### 2.3 Location groups

The SDI is a composite indicator developed by the Institute for Health Metrics and Evaluation as part of the GBD framework (22). It reflects a country’s development level based on lag-distributed income per capita, average educational attainment in the population aged ≥15 years, and total fertility rate among women under age 25. According to the GBD 2021 classification, 204 countries and territories were categorized into five SDI levels: low, low-middle, middle, high-middle, and high SDI.

For broader regional comparisons, the GBD study also grouped these countries into 21 predefined GBD regions based on a combination of geographic proximity and similarities in sociodemographic and epidemiological profiles. In this study, we adopted these standard SDI levels and GBD regional groupings without further modification to ensure consistency with GBD methodology and comparability with previous research (23).

### 2.4 Age groups

We categorized age groups in five-year intervals starting from 20 years onward. Specifically, women of reproductive age were divided into six groups: 20–24, 25–29, 30–34, 35–39, 40–44, and 45–49 years, to present disease burden indicators across each age category (24). This stratification enables age-specific analysis and helps reveal differences in disease burden throughout the reproductive lifespan.

### 2.5 Joinpoint regression analysis

Temporal trends in age-standardized mortality rates (ASMR) and disability-adjusted life year rates (ASDR; per 100,000 population) for OC and UC were assessed using Joinpoint Regression Program version 4.9.1 (25). Data obtained from the GBD database were processed and organized using Excel 2019 and R version 4.2.1 (26). The Joinpoint analysis applied a grid search method to detect potential trend change points, optimizing model fit by minimizing the sum of squared errors and evaluating mean squared errors for each configuration.

### 2.6 Model development and projection of future disease burden

Future age-standardized rates (ASRs) were projected through the year 2036 using a Bayesian age–period–cohort (BAPC) model (27). This approach accounts for temporal trends across age groups, time periods, and birth cohorts to estimate future disease burden with greater accuracy. The BAPC model decomposes the disease burden into three components: age effects, which capture variations in burden across different age groups; period effects, which reflect temporal influences such as healthcare improvements and policy changes; and cohort effects, which account for generational differences in disease risk. The model assumes a log-linear relationship between these effects and disease burden, with the effects modeled as random variables following a random walk process. For parameter estimation, Gaussian prior distributions were used, and Markov Chain Monte Carlo methods were applied to derive posterior distributions. Uncertainty in the projections was quantified using 95% UIs, generated from 1,000 posterior simulations, reflecting the variability in future disease burden projections.

### 2.7 Ethics statement

This study does not involve animal experiments, human participant research, or the use of identifiable human data or images.

### 2.8 Statistical analysis

Statistical analyses were conducted using R software (v4.2.2) or GraphPad Prism V.9. Details of the specific statistical tests applied are provided in the figure legends. A p-value < 0.05 was considered statistically significant.

## 3 Results

### 3.1 Global burden in 2021

In 2021, OC attributable to high BMI resulted in 2,022 deaths and 99,915 DALYs among women of reproductive age worldwide. Similarly, UC associated with high BMI led to 2,202 deaths and 114,177 DALYs (**Supplementary Tables S1**, **S2**). In 2021, the ASRs per 100,000 people for OC were 0.10 (95% UI: 0.02–0.19) for deaths and 5.13 (95% UI: 1.20–9.16) for DALYs; for UC, the ASRs were 0.11 (95% UI: 0.08–0.15) for deaths and 5.86 (95% UI: 4.11–7.71) for DALYs across 204 countries and territories (**Supplementary Tables S1**, **S2**).

**Figure 1:**
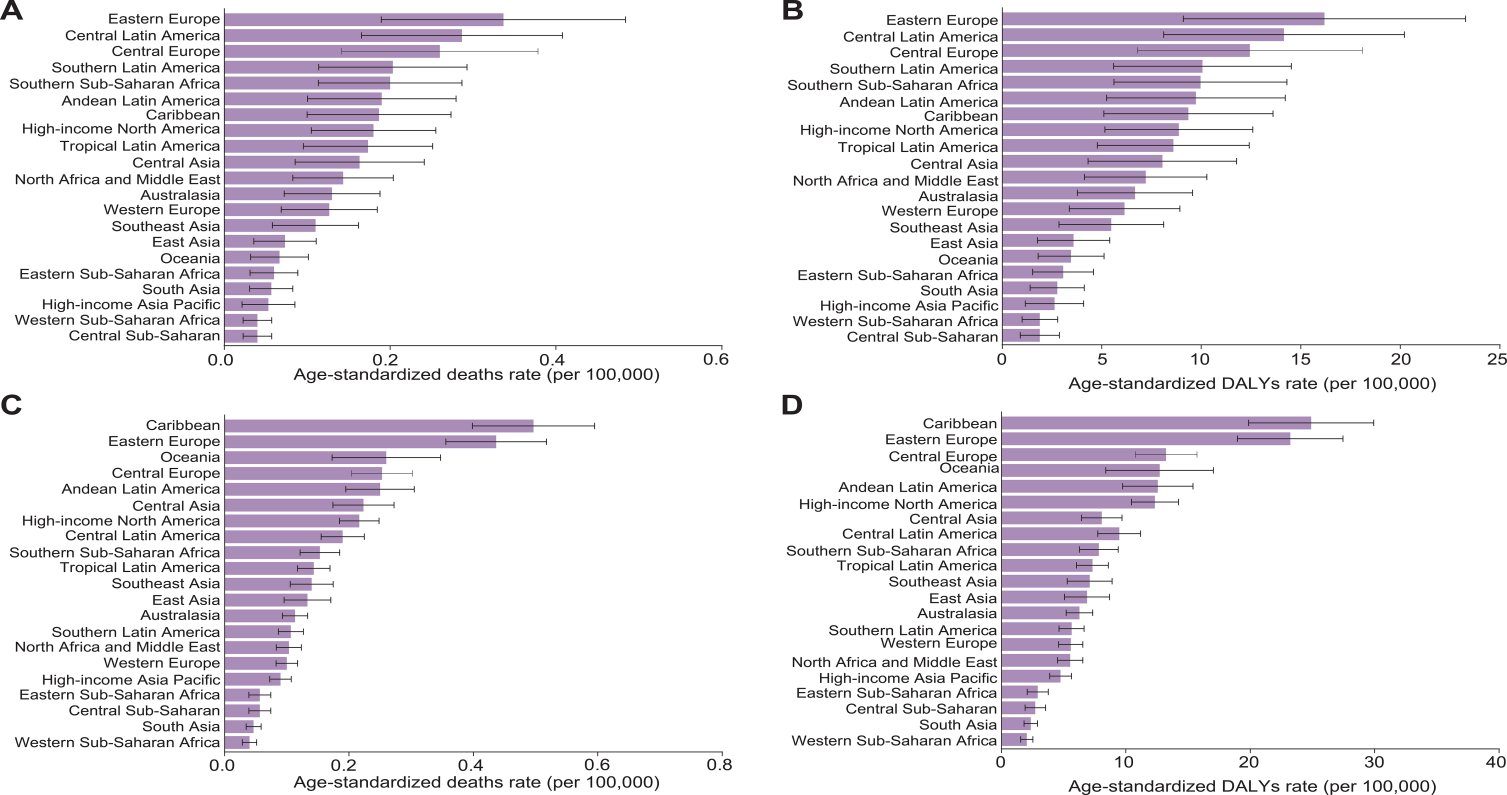
**(A–D)** The age-standardized death **(A, C)** and DALYs **(B, D)** rates attributable to high BMI for OC **(A, B)** and UC **(C, D)** among women of reproductive age in 2021 among 21 GBD regions. Data are presented as the mean ± Standard Error of the Mean (SEM). Error bars represent the 95% UIs. Data are visualized using bar charts to illustrate regional variations in disease burden.

**Figure 2:**
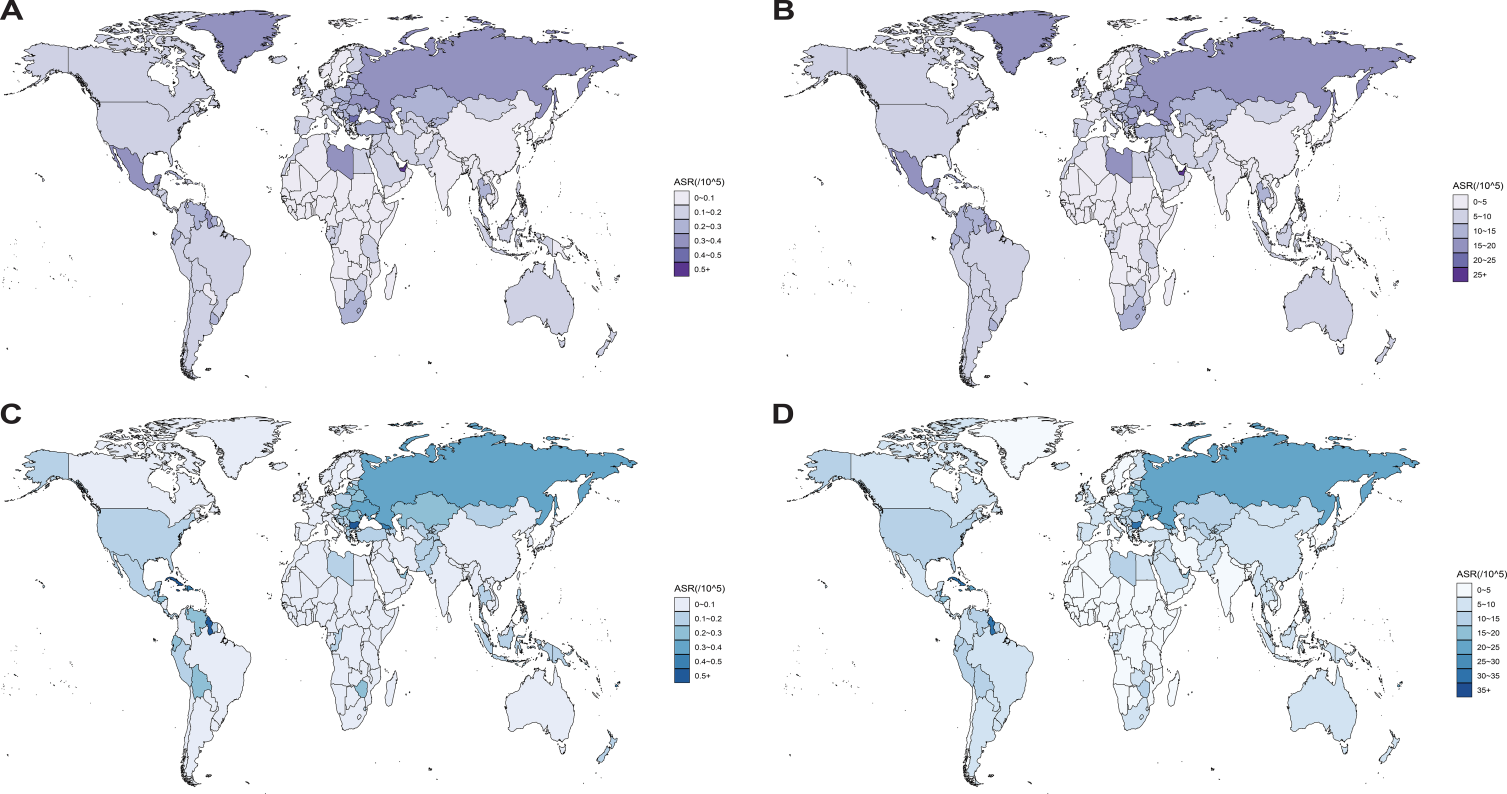
**(A–D)** The age-standardized death **(A, C)** and DALYs **(B, D)** rates attributable to high BMI for OC **(A, B)** and UC **(C, D)** per 100,000 population among women of reproductive age in 2021, by country and territory. Regional variations are illustrated using global heatmaps to highlight geographical disparities in disease burden.

**Figures 1**, **2** illustrate the global distribution of age-standardized deaths rates and DALYs for OC and UC attributable to high BMI among women of reproductive age in 2021.

For OC, Eastern Europe exhibited the highest age-standardized deaths rate (0.34 [95% UI, 0.08–0.59]) and DALYs (16.16 [95% UI, 3.92–28.48]). For UC, the Caribbean recorded the highest age-standardized deaths rate (0.49 [95% UI, 0.33–0.67]) and DALYs (24.53 [95% UI, 16.38–33.8]). The Bahamas recorded the highest ASRs among 204 countries and territories for deaths (0.55 [95% UI, 0.14–1.00]) and DALYs (27.34 [95% UI, 6.95–49.82]) in OC (**Supplementary Table S3**). The Northern Mariana Islands recorded the highest ASRs among 204 countries and territories for deaths (1.10 [95% UI, 0.66–1.70]) and DALYs (54.75 [95% UI, 32.89–84.16]) in UC (**Supplementary Table S4**).

### 3.2 Burden trends by age

**Figure 3:**
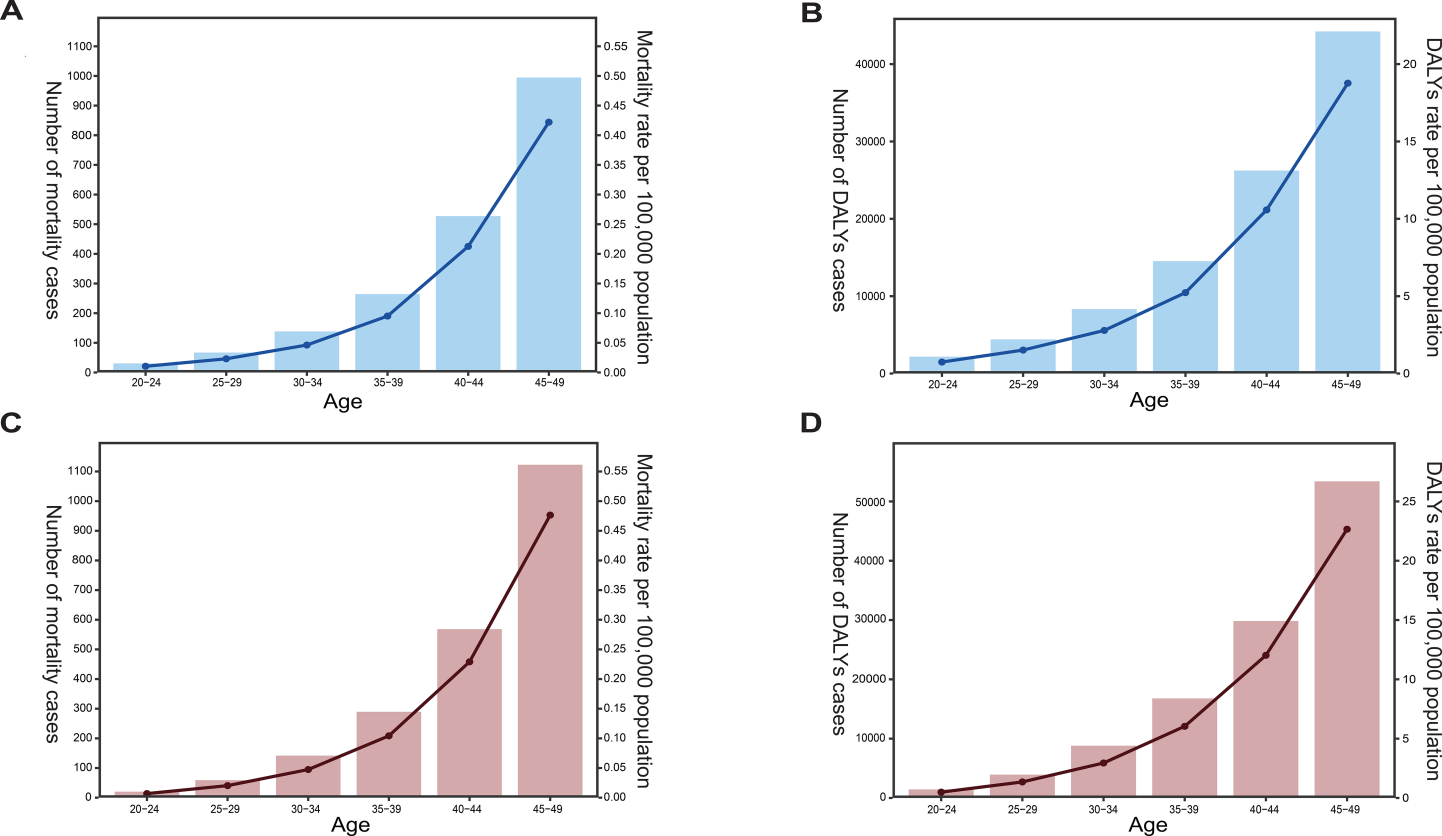
**(A–D)** The mortality rate and cases **(A, C)** or DALYs rate and cases **(B, D)** attributable to high BMI for OC **(A, B)** and UC **(C, D)** per 100,000 population among women across six age groups of reproductive age in 2021. The left y-axis in each panel represents the number of cases, while the right y-axis represents the corresponding rate. Trends across the six reproductive-age groups were illustrated using line graphs to depict temporal changes in disease burden indicators over ages.

Globally, the mortality rates and DALYs of OC and UC in women of reproductive age gradually increase with age, and the highest mortality rates and DALYs occur in the population aged 45-49. Specifically, the highest mortality rate and DALYs of OC were (0.42 [95% UI, 0.099-0.75]) and (4.42 [95% UI, 18.77-33.45]), respectively; the highest mortality rate and DALYs of UC were (0.48 [95% UI, 0.33-0.63]) and (22.66 [95% UI, 15.93-30.00]), respectively (**Figure 3**; **Supplementary Table S5**).

### 3.3 Different burden across SDI levels

From 1990 to 2021, the burden of OC and UC attributable to high BMI among women of reproductive age showed marked regional disparities influenced by the SDI.

**Figure 4:**
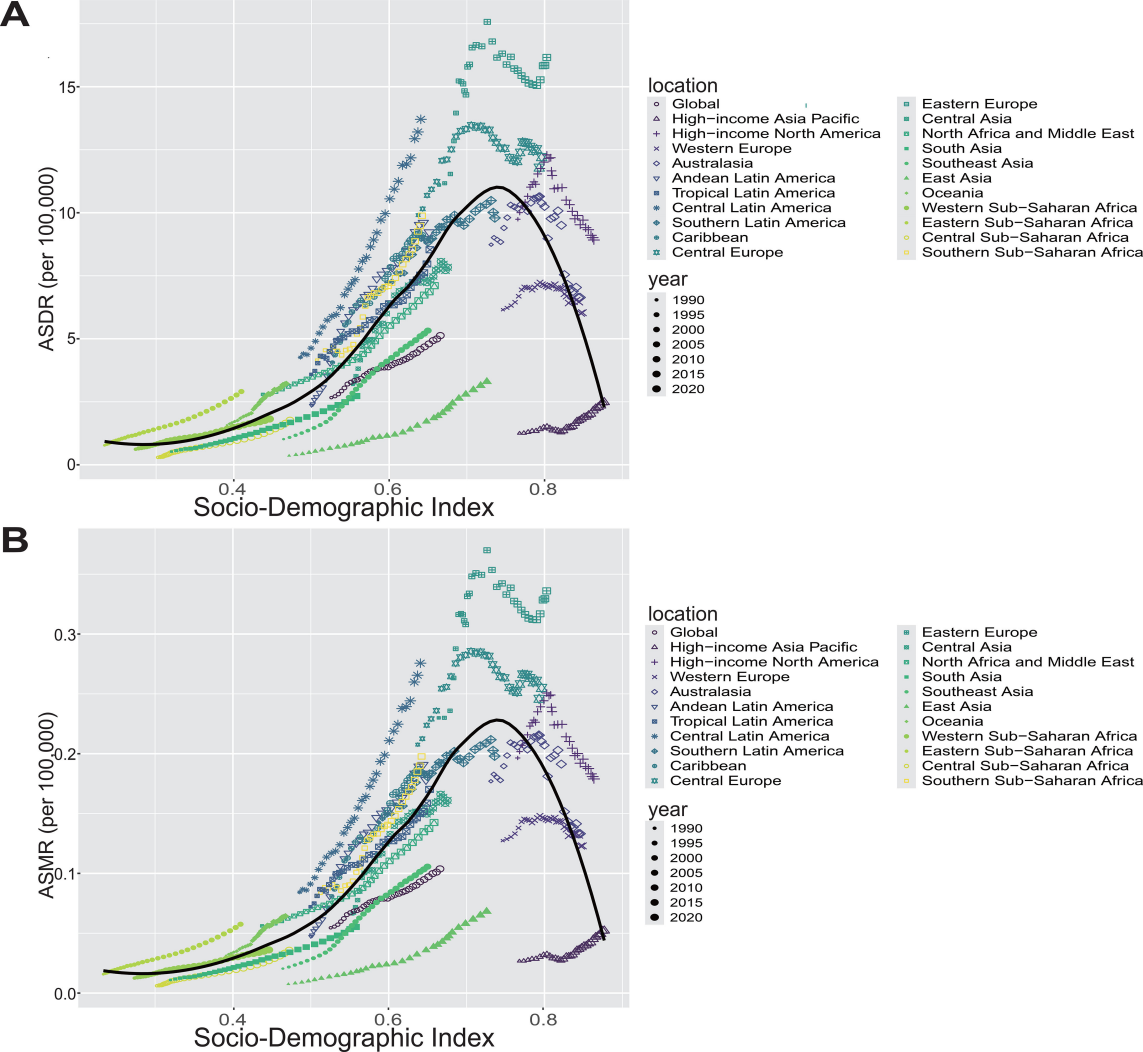
**(A, B)** The ASDR **(A)** and ASMR **(B)** of OC attributable to high BMI among women of reproductive age across 21 GBD regions by SDI from 1990 to 2021. For each region, points from left to right represent annual estimates for each year during the study period. Fitted curves illustrate the relationship and temporal trends between SDI and disease burden indicators.

Regarding OC, globally, the ASDR attributable to high BMI among women of reproductive age was consistently higher than the ASMR for the same condition, underscoring the substantial burden of disability and premature death. While global ASDR showed a slight downward trend, ASMR remained relatively stable and low, typically below 0.4 per 100,000. High SDI regions such as High-income Asia Pacific, Western Europe, and High-income North America, both ASDR and ASMR exhibited an upward trend in the early 2000s, followed by a sustained decline in ASDR and ASMR thereafter. Conversely, middle- and low-SDI regions, such as Central Asia, Eastern Europe, and Sub-Saharan Africa, showed increasing ASDR and ASMR trends (**Figures 4A, B**). Notably, the ASDR in these regions rose more steeply, suggesting a higher burden of early mortality.

As for UC, the overall trends in ASDR and ASMR across most regions were similar to those observed for OC. However, in high-SDI regions, both ASDR and ASMR generally showed an upward trend with increasing SDI levels. Notably, unlike OC, the ASMR for UC remained consistently below 0.6 per 100,000 across all regions (**Supplementary Figures 1A, B**). Analysis of the association between SDI and disease burden revealed a non-linear, inverted U-shaped relationship. The black curve peaked around the middle to upper-middle SDI range, where both ASDR and ASMR reached their highest levels; beyond this point, these rates declined as SDI increased further. This pattern suggests that regions with middle SDI levels experience increasing disease burden possibly due to rising exposure to risk factors coupled with limited health system capacity. Conversely, high SDI regions benefit from advanced healthcare infrastructure and public health interventions that effectively reduce disease burden (**Supplementary Table S6**). Additionally, high-middle SDI regions had the highest burden in terms of ASDR and DALYs for both cancers, while the low SDI region had the lowest ASDR and DALYs (**Supplementary Tables S1**, **S2**). Furthermore, our subgroup analysis by SDI categories showed that OC burden was significantly higher in low and middle SDI countries compared to high SDI countries, as reflected by elevated ASRs (P < 0.0001). In contrast, UC burden did not differ significantly across SDI groups (P > 0.05), indicating distinct epidemiological patterns between these two gynecologic cancers (**Supplementary Figures 2A, B**).

### 3.4 Global trends from 1990 to 2021

**Figure 5:**
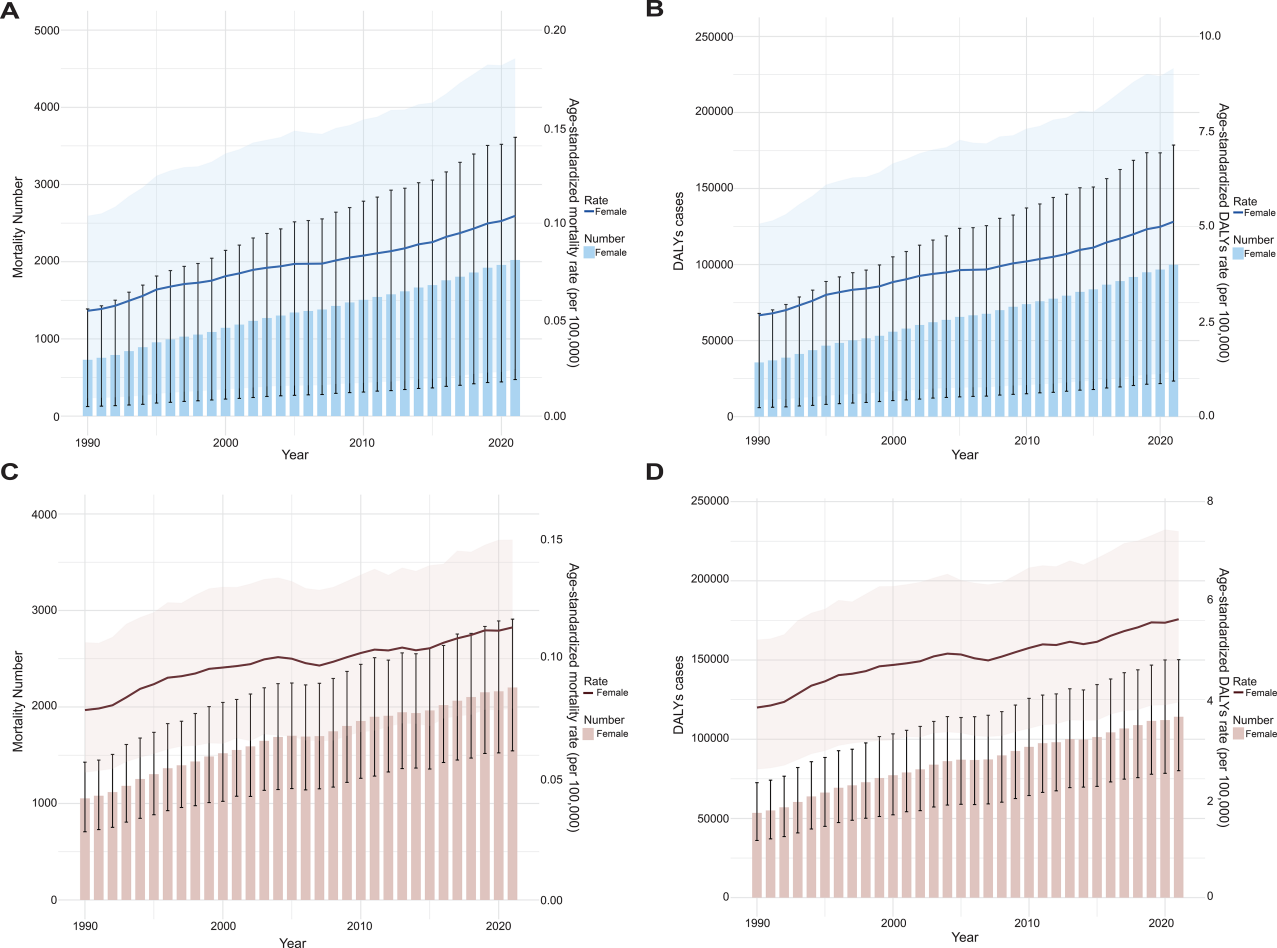
The trend analysis of ASRs of mortality **(A, C)** and DALYs **(B, D)** of OC and UC attributable to high BMI for global OC **(A, B)** and UC **(C, D)** among women of reproductive age (per 100–000 population) from 1990 to 2021. Data are presented as the mean ± SEM. Error bars represent the 95% UIs. Data are visualized using bar charts to illustrate variations in disease burden from 1990 to 2021.

From 1990 to 2021, the ASRs of mortality and DALYs for OC attributable to high BMI among women of reproductive age relatively increased, with mortality rising from 0.055 [95% CI, 0.0093–0.1] to 0.10 [95% CI, 0.024–0.19]), and DALYs increasing from 2.67 [95% CI, 0.44–5.08] to 5.13 [95% CI, 1.2–9.16]) (**Figures 5A, B**). As expected, similar trends were observed for UC, from 1990 to 2021, the age-standardized mortality rate and DALYs of UC due to high BMI in women of reproductive age showing relative increases over the same period. Specifically, mortality rose from 0.079 [95% CI, 0.053–0.11] to 0.11 [95% CI, 0.079–0.15]), while DALYs increased from 4 (95% CI: 2.7–5.43) to 5.86 (95% CI: 4.11–7.71) (**Figures 5C, D**).

From 1990 to 2021, among the 21 geographical regions, East Asia exhibited the largest increases in both mortality and DALYs for OC, with estimated annual percentage changes (EAPCs) of 7.20 (95% CI, 7.00–7.41) and 7.22 (95% CI, 7.02–7.41), respectively. Similarly, Southeast Asia showed the greatest rise in these indicators for UC, with EAPCs of 3.09 (95% CI, 2.84–3.35) for mortality and 3.07 (95% CI, 2.81–3.32) for DALYs (**Supplementary Tables S1**, **S2**). In contrast, Australasia (OC) and Southern Latin America (UC) demonstrated the most significant declines, with EAPCs of –1.00 and –0.97 for OC, and –1.28 and –1.16 for UC, respectively (**Supplementary Tables S1**, **S2**). At the SDI level, low-middle SDI regions showed the most pronounced increases in mortality and DALYs for both cancers, while high SDI regions experienced marked declines (**Supplementary Tables S1**, **S2**). To further investigate whether the observed regional disparities in the EAPCs are related to sociodemographic development, we found that regions with high SDI levels exhibited significantly lower EAPCs in OC attributable to high BMI than those with lower SDI levels (P < 0.05), suggesting a potential inverse association between SDI and the burden trend of OC (**Supplementary Figure 3A**). In contrast, no clear pattern or statistically significant difference was observed among SDI levels for UC (P > 0.05), indicating that the association between SDI and EAPC may differ by cancer type (**Supplementary Figure 3B**).

To further elucidate the global patterns beyond regional and SDI-level disparities, we next examined the trends in mortality and DALYs attributable to high BMI for OC and UC among women of reproductive age across 204 individual countries and territories from 1990 to 2021.

**Figure 6:**
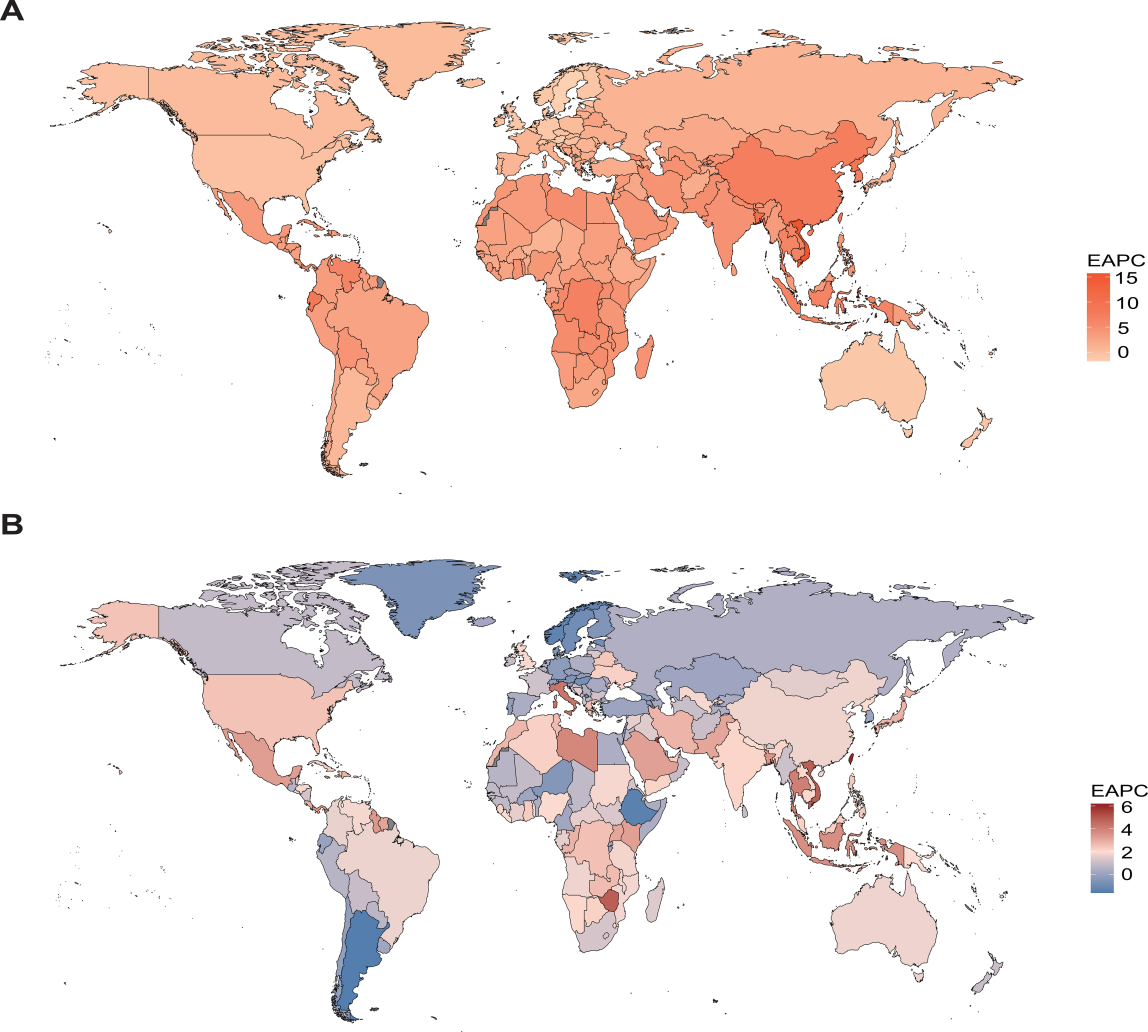
EAPCs of mortality attributable to high BMI among women of reproductive age in 2021, by country and territory. The regional differences in the EAPCs of mortality for OC **(A)** and UC **(B)** are displayed, visualized using global heatmaps.

At the national level, Vietnam reported the sharpest increases in both the mortality and DALY rates for OC, with EAPCs of 15.72 and 14.14, respectively. This was followed by Bangladesh, which had the second highest EAPCs in mortality and DALYs, at 10.43 and 10.46. In the case of UC, Taiwan (Province of China) experienced the most notable upward trends, showing EAPCs of 6.19 for mortality and 6.25 for DALYs. On the other hand, Sweden showed the greatest decline for OC, with EAPCs of –1.80 and –1.70, while Argentina had the most marked reductions for UC, with corresponding EAPCs of –1.63 and –1.52 (**Figure 6**; **Supplementary Figure 4**; **Supplementary Tables S3**, **S4**).

**Figure 7:**
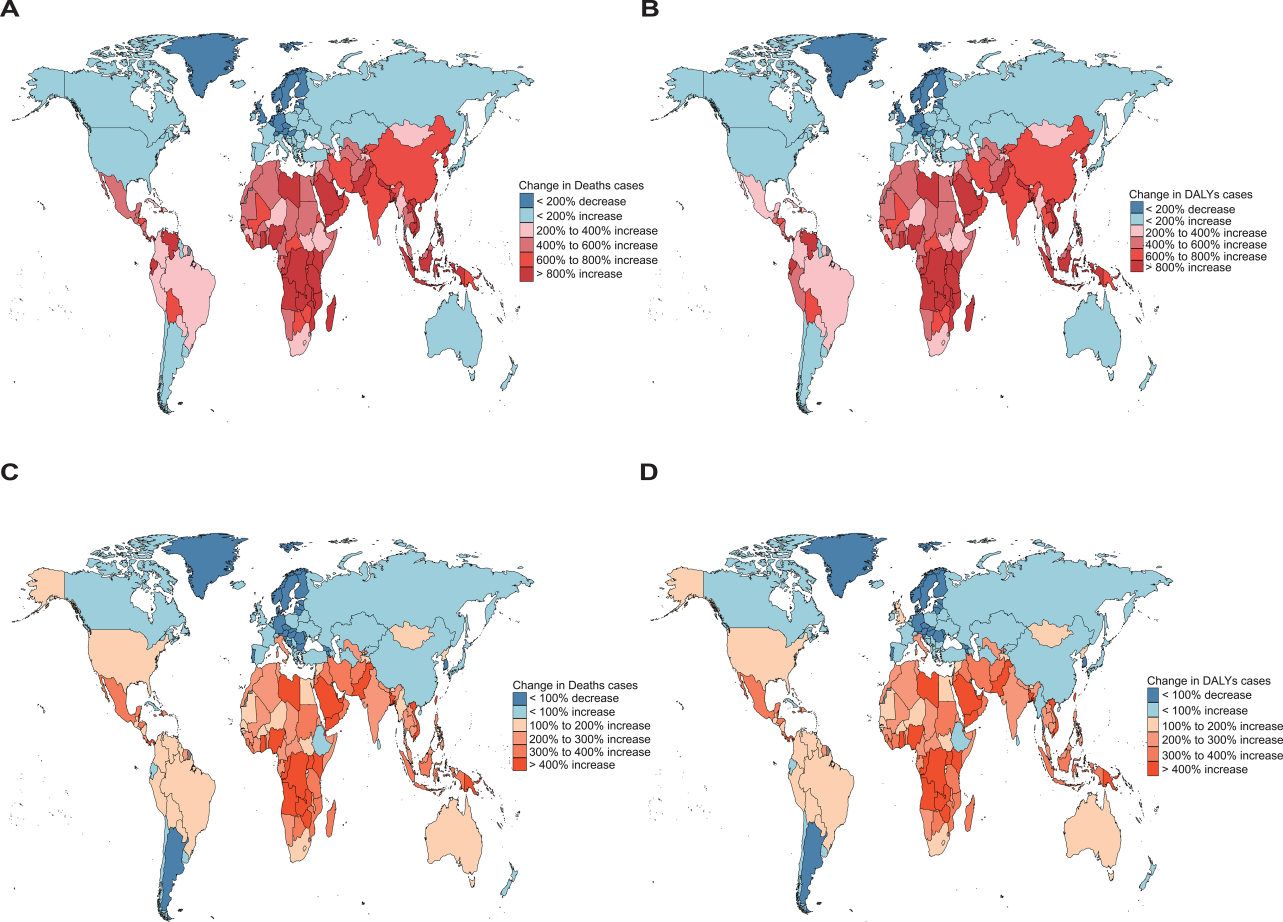
Absolute number changes in deaths and DALYs attributable to high BMI among women of reproductive age from 1990 to 2021, with **(A, B)** showing OC [**(A)**: deaths; **(B)**: DALYs] and panels **(C, D)** showing UC [**(C)**: deaths; **(D)**: DALYs]. Regional variations are illustrated using global heatmaps to highlight geographical disparities in disease burden. Geographic disparities in absolute number changes in deaths and DALYs were highlighted through the use of world heatmaps, which depict regional variation across countries and territories.

In addition to the trends in ASRs, we also evaluated the absolute number change in deaths and DALYs attributable to high BMI from 1990 to 2021, which reflects the overall burden influenced by population growth and aging. Regarding OC, Vietnam exhibited the largest increase in the number of deaths, with a percentage change of 13,713.69%, while Estonia showed the greatest decrease, with a decline of 42.73%. A similar pattern was observed for DALYs, with the highest increase also in Vietnam (8,928.14%) and the most significant decrease in Estonia (−42.26%) (**Figures 7A, B**; **Supplementary Tables S7**, **S8**). For UC, Kuwait experienced the largest rise in death numbers (1,761.86%), whereas Georgia showed the greatest reduction (−47.56%). In terms of DALYs, the largest increase was again in Kuwait (1,880.86%), and the greatest decrease was observed in Georgia (−47.50%) (**Figures 7C, D**; **Supplementary Tables S7**, **S8**).

### 3.5 Projections of the global trends to 2036

**Figure 8:**
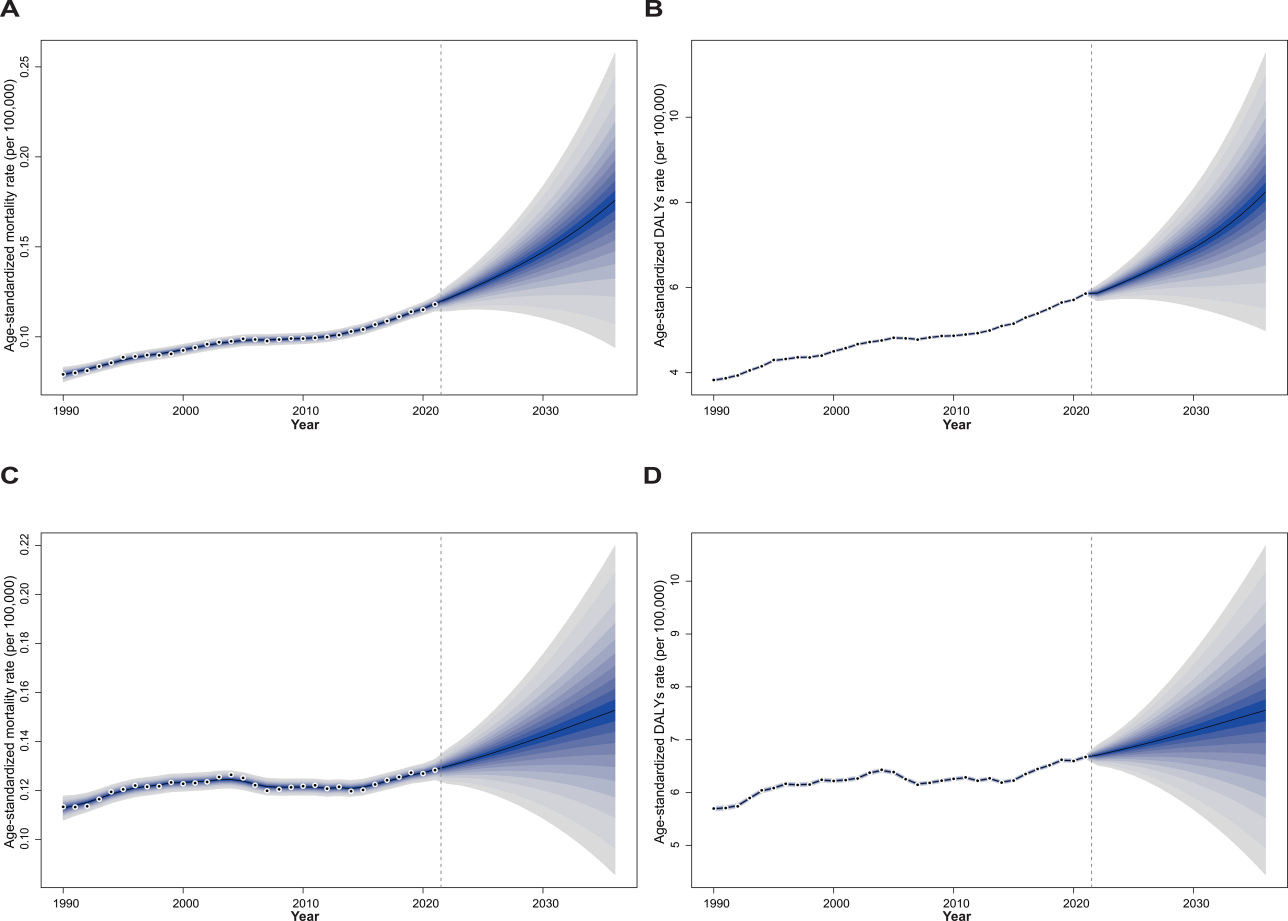
The trends of age-standardized numbers of deaths and DALYs attributable to high BMI among women of reproductive age for OC and UC from 2022 to 2036 predicted by Bayesian age-period-cohort model. Panels **(A, B)** show OC, and panels **(C, D)** show UC; panels **(A, C)** present deaths, while panels **(B, D)** present DALYs. Blue shades represent the corresponding confidence intervals.

As shown in **Figure 7**, we project a slight increase in the death rates and DALYs of OC and UC attributable to high BMI among women of reproductive age over the next 15 years. Specifically, the death rate of OC is expected to rise modestly from 0.12 per 100,000 in 2021 to 0.18 per 100,000 in 2036, while the DALYs are projected to increase from 5.86 per 100,000 in 2021 to 8.25 per 100,000 in 2030 (**Figures 8A, B**). For UC, the death rate is estimated to increase slightly from 0.13 per 100,000 in 2021 to 0.15 per 100,000 in 2036, and DALYs are projected to rise from 6.67 per 100,000 in 2021 to 7.56 per 100,000 in 2036 (**Figures 8C, D**).

## 4 Discussion

Our analysis highlights a growing global burden of OC and UC attributable to high BMI among women of reproductive age. In 2021 alone, high BMI contributed to over 4,200 deaths and more than 214,000 DALYs globally for these two gynecologic cancers. Although the overall age-standardized mortality and DALY rates appear relatively low, the trends over the past three decades reveal a worrying increase, particularly in low- and middle-SDI regions. Although these rates are relatively low in absolute terms, their upward trends over the study period highlight a growing and preventable burden, warranting greater public health attention. This underscores the expanding impact of obesity as a modifiable risk factor and the need for tailored public health strategies.

Regional disparities were evident across all GBD super-regions, shaped by a complex interplay of socioeconomic, health system, and cultural factors. While some high-SDI regions such as High-income Asia Pacific and Western Europe experienced a decline in both ASDR and ASMR after the early 2000s—likely due to robust obesity control policies, widespread access to gynecologic screening, and well-established cancer care systems—low- and middle-SDI regions, especially in Eastern Europe, Central Asia, and Sub-Saharan Africa, witnessed persistent or rising burdens. These disparities may be attributed to slower implementation of obesity prevention initiatives, limited public awareness, and insufficient investment in women’s health services. The sharpest increases in OC and UC were observed in countries such as Timor-Leste, Taiwan (Province of China), and Vietnam, which may reflect the combined effects of rising obesity prevalence, rapid urbanization, and lifestyle transitions, including shifts toward high-calorie diets and sedentary behavior. Additionally, these countries may face barriers such as underdeveloped healthcare infrastructure, lack of routine gynecologic cancer screening, and delayed diagnosis, all of which can exacerbate the health impact of obesity. Conversely, countries like Sweden and Argentina demonstrated marked declines, likely attributable to sustained public health investments and improved gynecologic care.

Notably, the impact of high BMI appears to intensify with age, amplifying its effects in regions where health systems may already be strained. These patterns are further reinforced by our findings at the age level, where women aged 45–49 consistently bore the greatest burden for both cancers. This trend reflects the cumulative physiological impact of prolonged exposure to excess body weight, including insulin resistance, estrogen imbalance, and chronic low-grade inflammation, all of which are known to promote tumorigenesis in gynecologic tissues (28–31). The high burden among older reproductive-aged women highlights a critical intervention window, underscoring the importance of early prevention strategies that begin well before the onset of middle age.

Furthermore, the marked increase in absolute numbers of deaths and DALYs, especially in low- and middle-income countries, reflects not only the rising prevalence of high BMI but also demographic transitions such as population growth and aging. These factors place additional strain on already under-resourced health systems. For instance, the disproportionate increases observed in countries like Vietnam and Kuwait underscore the need for context-specific strategies that integrate obesity prevention with reproductive health and cancer control programs. Without substantial investment in health system capacity and population-level behavioral interventions, these trends are unlikely to reverse.

A key limitation of our study lies in the nature of the GBD 2021 framework, which does not allow for direct comparisons between individuals with high BMI and those with normal BMI. Specifically, the GBD Study estimates disease burden attributable to high BMI using population-level exposure distributions and relative risks rather than providing stratified outcomes for different BMI categories. As such, our analysis could not evaluate or contrast the burden metrics (e.g., DALYs, mortality) between individuals with high BMI and those within the normal BMI range. This constraint limits our ability to make direct group-level inferences or quantify disparities in disease outcomes across BMI categories. Future studies based on individual-level cohort data may help to complement our findings and provide more granular insights into how risks vary across the BMI spectrum.

Looking toward the future, projections to 2036 suggest a continued, albeit modest, increase in mortality and DALYs for both cancers if no additional interventions are implemented. While the burden may stabilize in some high-SDI regions, many lower-SDI countries are expected to face rising trends, possibly driven by ongoing urbanization, nutritional transitions, limited public health infrastructure, and inadequate integration of obesity prevention into national health agendas (32). In many such settings, cultural norms that may stigmatize gynecologic symptoms, restrict women’s access to care, or underemphasize preventive health behaviors may also contribute to diagnostic delays and treatment disparities.

To effectively address this growing challenge, comprehensive strategies are required. Integrating cancer prevention into existing non-communicable disease frameworks, strengthening BMI surveillance, and expanding access to gynecological health education and screening programs are critical steps (33, 34). Moreover, interventions should be culturally tailored, equity-oriented, and supported by cross-sector policies addressing upstream drivers of obesity such as food insecurity, urban planning, and physical activity infrastructure. Finally, international collaboration and sustained support for health system strengthening in vulnerable regions will be essential for reversing the rising trend of obesity-related OC and UC, thereby reducing premature mortality and disability among women globally (35, 36).

## 5 Conclusions

This study reveals a rising global burden of ovarian and uterine cancers attributable to high BMI among women of reproductive age, with significant disparities across regions and SDI levels. While some high-SDI regions show stabilization, many low- and middle-SDI areas face increasing trends. Without targeted interventions, this burden is projected to grow by 2036. Strengthening obesity prevention, early detection, and equitable healthcare access is essential to curb this trajectory.

## Data Availability

The original contributions presented in the study are included in the article/**Supplementary Material**. Further inquiries can be directed to the corresponding author/s.
