# Supplementary material for: Burden and trends of ovarian and uterine cancer due to high body mass index from 1990 to 2021: an age–period–cohort study based on the GBD 2021, and projections through 2036
Source: Front Oncol. 2025 Aug 22;15:1647757. doi: 10.3389/fonc.2025.1647757 (PMC12411177; doi:10.3389/fonc.2025.1647757)
Supplement: Supplementary file 1 [file DataSheet1.docx]

**This file includes:**

**Supplementary Figure S1-S4**

**Supplementary Table S1-S8**

**
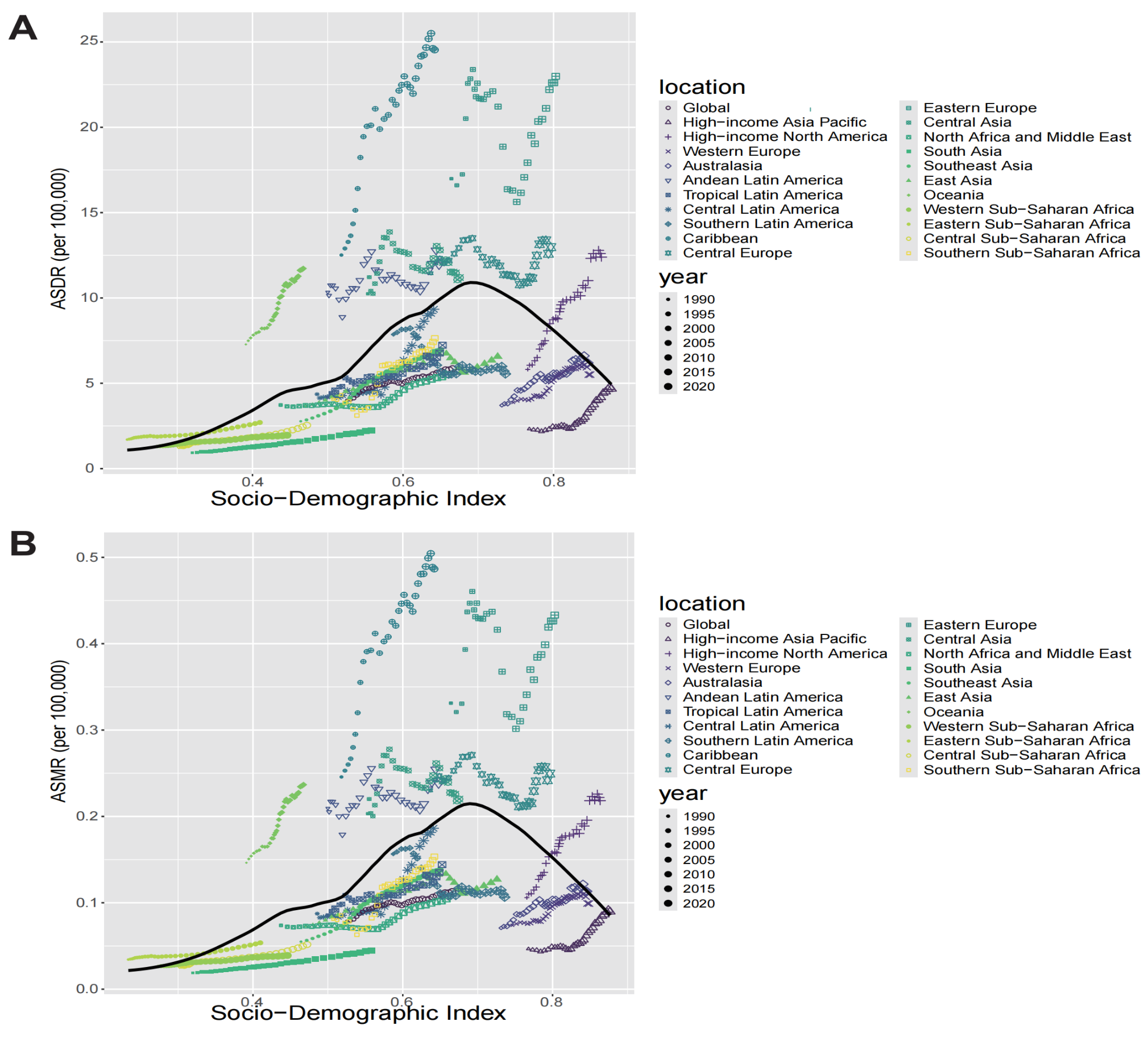
**

**Supplementary Fig. 1 (A-B) The ASDR (A) and ASMR (B) of UC attributable to high BMI among women of reproductive age across 21 GBD regions, stratified by SDI, from 1990 to 2021. Each point represents an annual estimate, from 1990 to 2021, for the respective region. Fitted curves highlight the trends and associations between SDI levels and the burden of UC.**

**
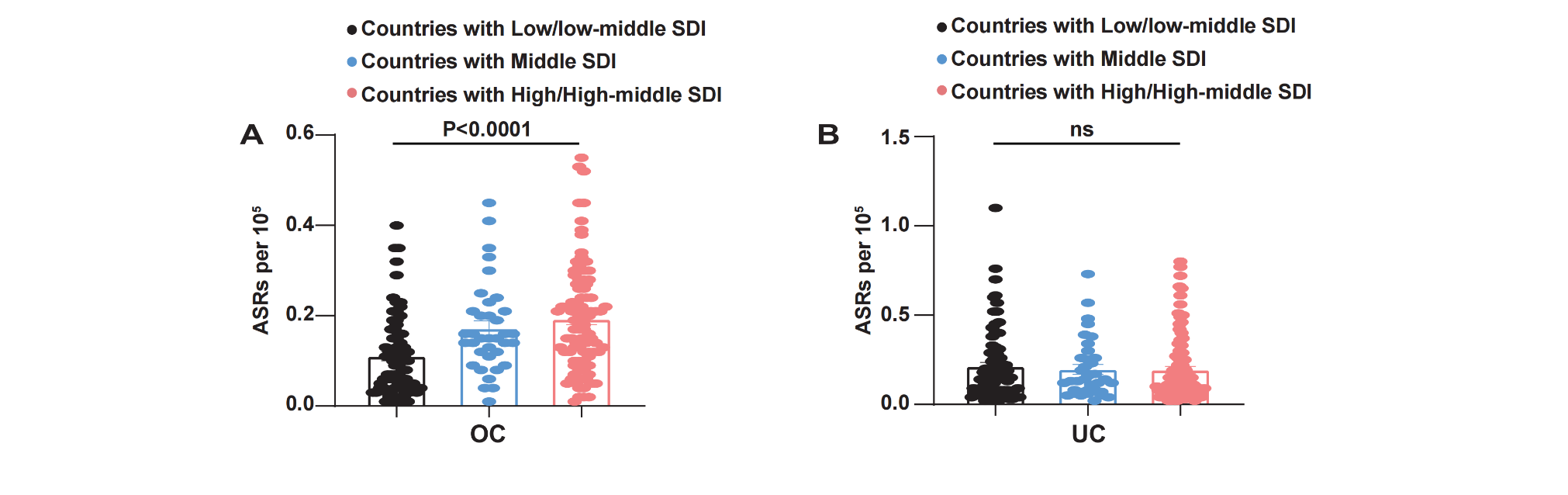
**

**Supplementary Fig. 2 Comparison of ASRs for OC (A) and UC (B) across different SDI categories. Countries were grouped into three SDI levels: high/high-middle, middle, and low/low-middle. ns, not significant. Data are presented as the mean ± SEM; one-way ANOVA followed by Dunnett's multiple comparisons test.**

**
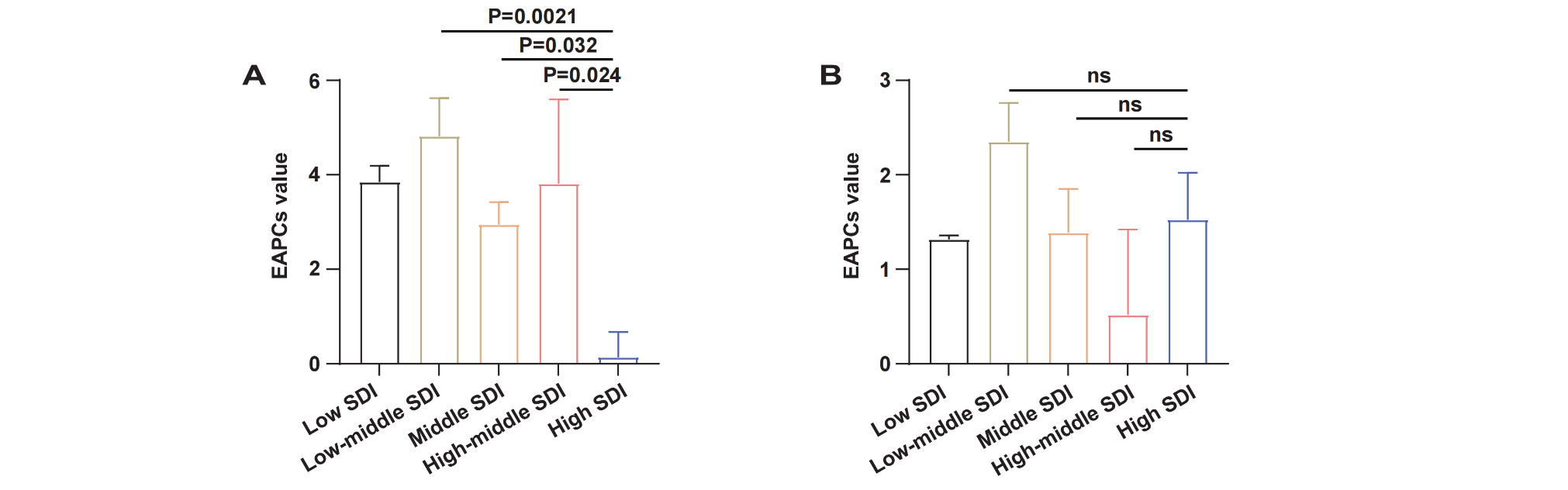
**

**Supplementary Fig. 3 (A-B) Comparison of EAPCs across different SDI levels in OC (A) and UC (B) attributable to high BMI, based on data from 21 GBD 2021 regions using EAPC estimates from 2021. Low SDI regions include Eastern Sub-Saharan Africa and Western Sub-Saharan Africa; Low-middle SDI regions are Central Sub-Saharan Africa, South Asia, Southeast Asia, and Tropical Latin America; Middle SDI regions include Andean Latin America, Central Latin America, the Caribbean, Central Asia, Eastern Europe, Oceania, and Southern Sub-Saharan Africa; High-middle SDI regions are East Asia, Southern Latin America, and North Africa and the Middle East; and High SDI regions include Australasia, Central Europe, High-income Asia Pacific, High-income North America, and Western Europe. A p-value of <0.05 was considered statistically significant. ns, not significant. Data are presented as the mean ± SEM; one-way ANOVA followed by Dunnett's multiple comparisons test.**

**
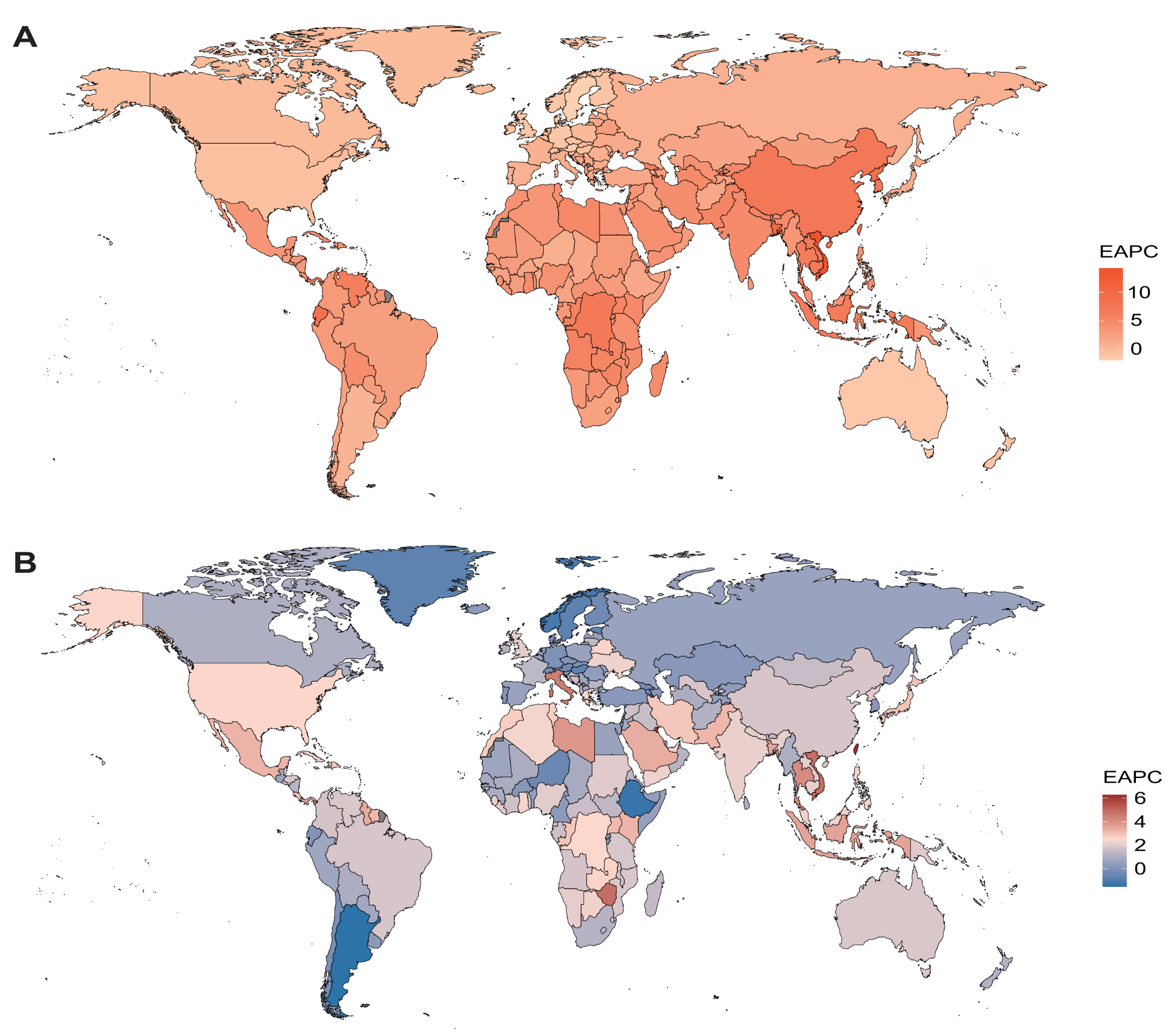
**

**Supplementary Fig. 4** EAPCs of DALYs attributable to high BMI among women of reproductive age in 2021, by country and territory. The regional variations in DALY EAPCs for OC (A) and UC (B) are shown, with global heatmaps providing a clear visualization of these disparities.

**Table S1 The age-standardized rate of deaths, and DALYs of OC attributable to high BMI in women of reproductive age in the world, 21 regions, and 5 SDI quintiles in 2021.**

| Location |  | Deaths |  |  | DALYs |  |
| --- | --- | --- | --- | --- | --- | --- |
|  | Number | ASRs (95% UI) | EAPC | Number | ASRs (95% UI) | EAPC |
| Global | 2022.06 | 0.1 (0.02-0.19) | 1.87 (1.76-1.98) | 99914.55 | 5.13 (1.2-9.16) | 1.91 (1.81-2.02) |
| **Region** |  |  |  |  |  |  |
| Andean Latin America | 32.02 | 0.18 (0.04-0.35) | 4.64 (4.11-5.18) | 1610.81 | 9.23 (2.22-17.74) | 4.68 (4.14 to 5.22) |
| Australasia | 9.61 | 0.13 (0.03-0.23) | -1 (-1.5 - -0.5) | 479.22 | 6.64 (1.67-11.7) | -0.97 (-1.46 to -0.48) |
| Caribbean | 22.13 | 0.18 (0.04-0.34) | 3.11 (2.77-3.44) | 1095.05 | 9.1 (2.12-16.84) | 3.03 (2.69 to 3.37) |
| Central Asia | 39.07 | 0.16 (0.03-0.3) | 2.85 (2.46-3.25) | 1901.59 | 7.84 (1.69-14.61) | 2.84 (2.47 to 3.22) |
| Central Europe | 65.66 | 0.25 (0.06-0.47) | 0.3 (0.01-0.6) | 3143.9 | 12.21 (2.78-22.35) | 0.34 (0.07 to 0.62) |
| Central Latin America | 187.97 | 0.28 (0.08-0.5) | 3.99 (3.91-4.07) | 9347.69 | 13.71 (3.91-24.85) | 3.93 (3.84 to 4.01) |
| Central Sub-Saharan | 11.68 | 0.04 (0.01-0.07) | 5.88 (5.75-6.01) | 577.21 | 1.77 (0.25-3.65) | 5.95 (5.82 to 6.07) |
| East Asia | 225.66 | 0.07 (0.01-0.14) | 7.2 (7-7.41) | 10913.17 | 3.3 (0.59-6.87) | 7.22 (7.02 to 7.41) |
| Eastern Europe | 162.12 | 0.34 (0.08-0.59) | 0.69 (0.31-1.07) | 7796.52 | 16.16 (3.92-28.48) | 0.69 (0.34 to 1.04) |
| Eastern Sub-Saharan Africa | 61.61 | 0.06 (0.01-0.11) | 4.19 (4.05-4.32) | 3113.34 | 2.91 (0.49 -5.78) | 4.29 (4.15 to 4.42) |
| High-income Asia Pacific | 19.83 | 0.05 (0-0.11) | 2.1(1.79-2.42) | 943.77 | 2.48 (0.16 -5.24) | 2.19 (1.9 to 2.49) |
| High-income North America | 150.32 | 0.18 (0.05-0.31) | -0.57 (-0.89 - -0.25) | 7493.23 | 8.92 (2.41-15.29) | -0.55 (-0.85 to -0.25) |
| North Africa and Middle East | 227.09 | 0.14 (0.04-0.25) | 3.09 (3.01-3.17) | 11324.08 | 7.11 (1.94-12.58) | 3.11 (3.03 to 3.19) |
| Oceania | 2.23 | 0.06 (0.01-0.13) | 2.36 (2.22-2.5) | 111.75 | 3.22 (0.72-6.44) | 2.33 (2.19-2.47) |
| South Asia | 273.06 | 0.06 (0.01-0.1) | 5.37 (5.32-5.43) | 13483.09 | 2.73 (0.43-5.14) | 5.49 (5.44-5.54) |
| Southeast Asia | 193.51 | 0.11 (0.02-0.2) | 5.6 (5.22-5.97) | 9753.34 | 5.32 (1.01-10.11) | 5.62 (5.24-6) |
| Southern Latin America | 34.51 | 0.2 (0.05-0.36) | 1.15 (0.99-1.32) | 1707.2 | 9.79 (2.47-17.93) | 1.22 (1.06-1.38) |
| Southern Sub-Saharan Africa | 42.92 | 0.2 (0.05-0.35) | 3.01 (2.84-3.18) | 2145.52 | 9.88 (2.48-17.52) | 2.9 (2.71-3.09) |
| Tropical Latin America | 103.15 | 0.17 (0.04-0.31) | 2.42 (2.3-2.53) | 5171.75 | 8.53 (2.02-15.24) | 2.45 (2.34-2.57) |
| Western Europe | 114.63 | 0.12 (0.03-0.23) | -0.15 (-0.36 - 0.05) | 5623.73 | 6.04 (1.39-11.01) | -0.11 (-0.32-0.09) |
| Western Sub-Saharan Africa | 43.26 | 0.04 (0.01-0.07) | 3.5 (3.44-3.56) | 2178.6 | 1.82 (0.38-3.48) | 3.55 (3.49-3.61) |
| **SDI** |  |  |  |  |  |  |
| Low SDI | 106.31 | 0.04 (0.01-0.08) | 4.05 (3.87-4.23) | 5325.32 | 1.94 (0.29-3.79) | 4.16 (3.99-4.33) |
| Low-middle SDI | 374.31 | 0.07 (0.02-0.13) | 5.1 (4.93-5.28) | 18705.52 | 3.69 (0.77-6.73) | 5.16 (4.98-5.34) |
| Middle SDI | 705.26 | 0.11 (0.03-0.21) | 4.58 (4.5-4.66) | 35002.97 | 5.66 (1.31-10.4) | 4.56 (4.48-4.64) |
| High-middle SDI | 482.56 | 0.16 (0.04-0.29) | 1.51 (1.31-1.71) | 23400.12 | 7.67 (1.75-14.01) | 1.52 (1.33-1.7) |
| High SDI | 351.17 | 0.14 (0.03-0.25) | -0.01 (-0.24-0.22) | 17360.72 | 7.14 (1.73-12.35) | 0.03 (-0.19-0.25) |

Table S2 The age-standardized rate of deaths, and DALYs of UC attributable to high BMI in women of reproductive age in the world, 21 regions, and 5 SDI quintiles in 2021.

| Location |  | Deaths |  |  | DALYs |  |
| --- | --- | --- | --- | --- | --- | --- |
|  | Number | ASR (95% UI) | EAPC | Number | ASR (95% UI) | EAPC |
| Global | 2201.8 | 0.11 (0.08-0.15) | 0.98 (0.86-1.1) | 114177.42 | 5.86 (4.11-7.71) | 1.05 (0.94-1.16) |
| **Region** |  |  |  |  |  |  |
| Andean Latin America | 42.19 | 0.24 (0.16-0.35) | 0.41 (0.15-0.66) | 2120.82 | 12.15 (7.9-17.68) | 0.46 (0.21-0.71) |
| Australasia | 8.18 | 0.11 (0.08-0.15) | 1.48 (1.27-1.68) | 447.05 | 6.19 (4.46-8.17) | 1.6 (1.39-1.81) |
| Caribbean | 58.52 | 0.49 (0.33-0.67) | 2.18 (1.85-2.51) | 2951.21 | 24.53 (16.38-33.8) | 2.11 (1.77-2.44) |
| Central Asia | 53.41 | 0.22 (0.14-0.31) | 0.09 (-0.24-0.42) | 2716.16 | 11.19 (7.27-5.75) | 0.08 (-0.22-0.38) |
| Central Europe | 64.28 | 0.25 (0.17-0.34) | -0.23 (-0.49-0.04) | 3342.24 | 12.98 (9.07-17.66) | -0.05 (-0.31-0.21) |
| Central Latin America | 127.04 | 0.19 (0.13-0.25) | 2.71 (2.24-3.17) | 6367.08 | 9.34 (6.56-12.55) | 2.72 (2.26-3.19) |
| Central Sub-Saharan | 16.9 | 0.05 (0.03-0.09) | 2.19 (2.07-2.31) | 830.12 | 2.54 (1.4-4.23) | 2.21 (2.09-2.34) |
| East Asia | 420.52 | 0.13 (0.07-0.2) | 1.61 (1.26-1.96) | 21724.62 | 6.57 (3.92-10.16) | 1.69 (1.36-2.03) |
| Eastern Europe | 208.93 | 0.43 (0.3-0.58) | -0.07 (-0.58-0.44) | 11089.66 | 22.99 (15.99-30.68) | 0.07 (-0.42-0.56) |
| Eastern Sub-Saharan Africa | 57.45 | 0.05 (0.03-0.09) | 1.27 (1.1-1.44) | 2893.83 | 2.7 (1.59-4.49) | 1.32 (1.15-1.49) |
| High-income Asia Pacific | 34.11 | 0.09 (0.06-0.12) | 2.54 (2.19-2.88) | 1778.27 | 4.67 (3.3-6.3) | 2.73 (2.41-3.05) |
| High-income North America | 183.38 | 0.22 (0.16-0.27) | 2.42 (2.25-2.59) | 10413.79 | 12.39 (9.04-15.59) | 2.53 (2.36-2.7) |
| North Africa and Middle East | 166.47 | 0.1 (0.07-0.14) | 1.22 (0.92-1.53) | 8774.26 | 5.51 (3.77-7.29) | 1.37 (1.07-1.68) |
| Oceania | 8.22 | 0.24 (0.12-0.42) | 1.61 (1.54-1.69) | 406.94 | 11.72 (5.77-20.67) | 1.58 (1.51-1.65) |
| South Asia | 220.86 | 0.04 (0.03-0.07) | 2.86 (2.76-2.96) | 11096.15 | 2.25 (1.49-3.34) | 2.93 (2.84-3.02) |
| Southeast Asia | 252.94 | 0.14 (0.08-0.2) | 3.09 (2.84-3.35) | 12739.89 | 6.95 (4.06-10.03) | 3.07 (2.81-3.32) |
| Southern Latin America | 18.56 | 0.11 (0.07-0.14) | -1.28 (-1.62- -0.95) | 967 | 5.55 (3.93-7.44) | -1.16 (-1.5- -0.83) |
| Southern Sub-Saharan Africa | 33.32 | 0.15 (0.1-0.21) | 2.77 (2.34-3.21) | 1658.37 | 7.64 (5.24-10.63) | 2.7 (2.24-3.17) |
| Tropical Latin America | 87.35 | 0.14 (0.1-0.19) | 1.25 (1.09-1.4) | 4382.35 | 7.23 (5.15-9.57) | 1.33 (1.17-1.49) |
| Western Europe | 92.39 | 0.1 (0.07-0.13) | 1.41 (1.2-1.62) | 5132.85 | 5.51 (3.92-7.3) | 1.6 (1.37-1.82) |
| Western Sub-Saharan Africa | 46.78 | 0.04 (0.02-0.06) | 1.36 (1.31-1.4) | 2344.75 | 1.96 (1.24-2.93) | 1.39 (1.35-.44) |
| **SDI** |  |  |  |  |  |  |
| Low SDI | 140.21 | 0.05 (0.03-0.08) | 1.59 (1.44-1.74) | 7022.07 | 2.56 (1.66-3.79) | 1.65 (1.51-1.79) |
| Low-middle SDI | 385.82 | 0.08 (0.05-0.1) | 2.23 (2.2-2.27) | 19396.58 | 3.83 (2.6-5.13) | 2.27 (2.24-2.3) |
| Middle SDI | 751.95 | 0.12 (0.08-0.17) | 1.79 (1.68-1.9) | 38134.99 | 6.17 (4.18-8.45) | 1.81 (1.71-1.91) |
| High-middle SDI | 555.22 | 0.18 (0.13-0.24) | 0.22 (-0.02-0.45) | 29261.03 | 9.59 (6.76-12.8) | 0.35 (0.13-0.56) |
| High SDI | 364.67 | 0.15 (0.11-0.19) | 1.79 (1.69-1.89) | 20162..72 | 8.29 (5.98-10.71) | 1.96 (1.86-2.06) |

Table S3 The age-standardized rate of deaths, and DALYs of OC attributable to high BMI in women of reproductive age in 204 countries and territories in 2021.

| Country or territories | Deaths | |  | DALYs | |  |  |
| --- | --- | --- | --- | --- | --- | --- | --- |
|  | Number | ASR (95% UI) | EAPC | Number | ASR (95% UI) | EAPC | SDI Quintile |
| Afghanistan | 5.05 | 0.07(0.01-0.21) | 1.9 | 249.7 | 3.47(0.59-10.44) | 2.02 | Low SDI |
| Albania | 0.47 | 0.08(0.02-0.16) | 2.82 | 22.94 | 3.74(0.75-7.76) | 2.78 | Middle SDI |
| Algeria | 8.69 | 0.08(0.02-0.15) | 3.73 | 431.54 | 3.85(1.03-7.33) | 3.76 | Middle SDI |
| American Samoa | 0.03 | 0.3(0.08-0.58) | 3.59 | 1.75 | 14.94(4.03-28.7) | 3.48 | High-middle SDI |
| Andorra | 0.01 | 0.05(0.01-0.12) | 1.42 | 0.52 | 2.61(0.5-5.61) | 1.38 | High SDI |
| Angola | 2.89 | 0.04(0-0.09) | 5.6 | 142.71 | 1.86(0.23-4.41) | 5.71 | Low-middle SDI |
| Antigua and Barbuda | 0.06 | 0.27(0.07-0.48) | 2.79 | 3.22 | 13.34(3.3-23.44) | 2.65 | High-middle SDI |
| Argentina | 23.7 | 0.2(0.05-0.37) | 0.63 | 1174.4 | 9.88(2.35-18.24) | 0.68 | High-middle SDI |
| Armenia | 1.15 | 0.16(0.04-0.29) | 1.69 | 55.28 | 7.5(1.66-13.82) | 1.67 | Middle SDI |
| Australia | 8.11 | 0.13(0.03-0.24) | -0.96 | 404.38 | 6.71(1.73-11.86) | -0.92 | High SDI |
| Austria | 1.64 | 0.08(0.02-0.15) | -1.32 | 79.91 | 4.05(0.76-7.47) | -1.21 | High SDI |
| Azerbaijan | 3.88 | 0.14(0.03-0.31) | 2.75 | 188.89 | 6.89(1.6-14.95) | 2.73 | Middle SDI |
| Bahamas | 0.59 | 0.55(0.14-1) | 2.63 | 29.47 | 27.34(6.95-49.82) | 2.54 | High-middle SDI |
| Bahrain | 1.02 | 0.31(0.09-0.61) | 3.17 | 51.15 | 15.69(4.61-30.04) | 3.05 | High-middle SDI |
| Bangladesh | 22.8 | 0.05(0.01-0.11) | 10.43 | 1161.29 | 2.52(0.37-5.83) | 10.46 | Low-middle SDI |
| Barbados | 0.27 | 0.38(0.12-0.69) | 1.98 | 13.41 | 18.84(5.67-33.78) | 1.88 | High-middle SDI |
| Belarus | 5.86 | 0.28(0.06-0.54) | 2.97 | 278.22 | 13.12(2.61-25.95) | 3.02 | High-middle SDI |
| Belgium | 3 | 0.12(0.03-0.23) | -0.32 | 147.86 | 5.97(1.25-11.08) | -0.28 | High SDI |
| Belize | 0.17 | 0.14(0.04-0.24) | 2.7 | 8.63 | 7.14(2.21-12.18) | 2.64 | Low-middle SDI |
| Benin | 0.97 | 0.03(0.01-0.06) | 1.88 | 48.65 | 1.5(0.31-3.19) | 1.96 | Low SDI |
| Bermuda | 0.05 | 0.39(0.11-0.69) | 1.48 | 2.6 | 19.3(5.65-34.32) | 1.43 | High SDI |
| Bhutan | 0.18 | 0.09(0.02-0.22) | 3.99 | 9.45 | 4.57(0.89-11.1) | 4.08 | Low-middle SDI |
| Bolivia (Plurinational State of) | 4.83 | 0.16(0.03-0.31) | 4.53 | 242.5 | 7.78(1.64-15.79) | 4.59 | Low-middle SDI |
| Bosnia and Herzegovina | 1.41 | 0.19(0.04-0.38) | 2.26 | 66.94 | 9.15(2-18.12) | 2.26 | High-middle SDI |
| Botswana | 0.96 | 0.14(0.03-0.29) | 4.16 | 49.5 | 7.28(1.55-14.89) | 4.29 | Middle SDI |
| Brazil | 101 | 0.17(0.04-0.31) | 2.82 | 5073.17 | 8.64(2.05-15.43) | 2.84 | High-middle SDI |
| Brunei Darussalam | 0.33 | 0.27(0.06-0.52) | 4.5 | 16.71 | 13.38(2.86-25.6) | 4.49 | High-middle SDI |
| Bulgaria | 5.79 | 0.41(0.1-0.77) | 1.58 | 277.01 | 19.45(4.56-37.25) | 1.58 | High-middle SDI |
| Burkina Faso | 0.53 | 0.01(0-0.02) | 2.14 | 26.23 | 0.48(0-1.15) | 2.16 | Low SDI |
| Burundi | 0.41 | 0.01(0-0.04) | 3.85 | 21.09 | 0.67(-0.13-1.93) | 3.96 | Low SDI |
| Cabo Verde | 0.06 | 0.04(0.01-0.09) | 6.64 | 3.31 | 2.21(0.49-4.45) | 6.67 | Low-middle SDI |
| Cambodia | 2.32 | 0.05(0-0.12) | 6.7 | 115.46 | 2.56(0.06-6.2) | 6.83 | Low-middle SDI |
| Cameroon | 4.15 | 0.05(0.01-0.11) | 2.18 | 212.65 | 2.71(0.55-5.39) | 2.3 | Low-middle SDI |
| Canada | 11.8 | 0.14(0.03-0.25) | 0.15 | 585.46 | 7.06(1.7-12.46) | 0.18 | High SDI |
| Central African Republic | 0.44 | 0.03(0-0.07) | 3.9 | 21.85 | 1.58(0.23-3.52) | 3.96 | Low SDI |
| Chad | 0.3 | 0.01(0-0.02) | 2.04 | 14.77 | 0.38(0.02-0.92) | 2.08 | Low SDI |
| Chile | 9.04 | 0.19(0.05-0.34) | 2.33 | 448.12 | 9.51(2.54-16.99) | 2.4 | High-middle SDI |
| China | 217 | 0.07(0.01-0.14) | 7.34 | 10478.98 | 3.29(0.6-6.89) | 7.35 | High-middle SDI |
| Colombia | 26.8 | 0.2(0.05-0.39) | 3.44 | 1343.76 | 10.26(2.35-19.26) | 3.49 | Middle SDI |
| Comoros | 0.24 | 0.13(0.02-0.27) | 5.19 | 12.32 | 6.31(1.08-13.72) | 5.23 | Low-middle SDI |
| Congo | 1.63 | 0.11(0.02-0.22) | 5.74 | 81.72 | 5.7(1.13-11.29) | 5.91 | Low-middle SDI |
| Cook Islands | 0 | 0.12(0.04-0.23) | 2.26 | 0.26 | 6.08(1.81-11.78) | 2.25 | High-middle SDI |
| Costa Rica | 2.41 | 0.19(0.04-0.33) | 4.41 | 121.6 | 9.39(2.25-16.83) | 4.4 | Middle SDI |
| Croatia | 4.28 | 0.24(0.05-0.45) | 1.23 | 216.05 | 11.48(2.53-21.66) | 1.28 | High-middle SDI |
| Cuba | 2.13 | 0.23(0.06-0.43) | 4.45 | 103.02 | 11.13(2.73-20.8) | 4.37 | Middle SDI |
| Cyprus | 5.73 | 0.12(0.02-0.25) | 2.37 | 277.08 | 5.7(1.21-12.13) | 2.43 | High SDI |
| Czechia | 0.41 | 0.21(0.05-0.39) | -0.31 | 20.35 | 9.85(2.27-18.78) | -0.26 | High SDI |
| Côte d'Ivoire | 4.7 | 0.06(0.01-0.12) | 2.76 | 226.01 | 3.24(0.66-6.43) | 2.83 | Low-middle SDI |
| Democratic People's Republic of Korea | 0.48 | 0.01(-0.01-0.04) | 9.23 | 24.52 | 0.37(-0.54-1.82) | 8.9 | Low-middle SDI |
| Democratic Republic of the Congo | 5.65 | 0.03(0-0.06) | 6.93 | 278 | 1.3(0.13-2.89) | 7.01 | Low SDI |
| Denmark | 1.11 | 0.09(0.02-0.16) | -1.17 | 54.49 | 4.31(0.95-7.84) | -1.04 | High SDI |
| Djibouti | 0.12 | 0.04(0-0.1) | 5.11 | 6.22 | 1.94(0.03-4.79) | 5.12 | Low-middle SDI |
| Dominica | 0.02 | 0.17(0.05-0.33) | 2.83 | 1.42 | 8.63(2.37-16.34) | 2.84 | High-middle SDI |
| Dominican Republic | 3.09 | 0.11(0.02-0.21) | 3.85 | 155.51 | 5.37(1.07-10.57) | 3.86 | Middle SDI |
| Ecuador | 9.78 | 0.21(0.05-0.38) | 8.68 | 494.39 | 10.48(2.58-19.21) | 8.69 | Middle SDI |
| Egypt | 34.7 | 0.13(0.04-0.25) | 3.92 | 1758.42 | 6.78(2-12.42) | 3.98 | Low-middle SDI |
| El Salvador | 4.03 | 0.23(0.06-0.45) | 3.91 | 202.32 | 11.39(3.08-22.3) | 3.91 | Low-middle SDI |
| Equatorial Guinea | 0.33 | 0.09(0.02-0.2) | 4.93 | 16.99 | 4.66(0.9-10.22) | 5.14 | Middle SDI |
| Eritrea | 0.38 | 0.02(0-0.06) | 4.03 | 18.95 | 1.14(-0.01-3.01) | 4.16 | Low SDI |
| Estonia | 0.57 | 0.21(0.05-0.37) | -0.78 | 27.87 | 10.01(2.25-18.2) | -0.76 | High SDI |
| Eswatini | 0.65 | 0.21(0.04-0.49) | 2.93 | 33.53 | 10.64(2.06-24.85) | 2.97 | Low-middle SDI |
| Ethiopia | 5.19 | 0.02(0-0.04) | 1.75 | 257.84 | 0.93(-0.06-2.1) | 1.85 | Low SDI |
| Fiji | 0.31 | 0.14(0.04-0.26) | 1.98 | 15.65 | 6.85(1.98-12.95) | 1.96 | Middle SDI |
| Finland | 1.27 | 0.11(0.03-0.22) | -0.73 | 63.56 | 5.59(1.31-10.79) | -0.63 | High SDI |
| France | 12.2 | 0.09(0.02-0.16) | 0.95 | 611.58 | 4.31(0.97-8.04) | 1.02 | High SDI |
| Gabon | 0.71 | 0.15(0.04-0.31) | 3.43 | 35.94 | 7.34(1.78-15.48) | 3.48 | Middle SDI |
| Gambia | 0.2 | 0.03(0.01-0.07) | 3.62 | 10.3 | 1.67(0.34-3.56) | 3.7 | Low SDI |
| Georgia | 2.63 | 0.33(0.08-0.63) | 5.68 | 127.48 | 16.2(3.63-30.44) | 5.75 | High SDI |
| Germany | 21.6 | 0.13(0.03-0.23) | -0.89 | 1067.83 | 6.27(1.45-11.42) | -0.82 | High SDI |
| Ghana | 5.68 | 0.06(0.01-0.14) | 4.63 | 289.92 | 3.18(0.66-6.98) | 4.68 | Low-middle SDI |
| Greece | 4.38 | 0.2(0.05-0.38) | 2.25 | 209.7 | 9.67(2.2-17.81) | 2.2 | High-middle SDI |
| Greenland | 0.04 | 0.32(0.07-0.62) | 0.25 | 1.96 | 15.41(3.4-30.19) | 0.09 | High SDI |
| Grenada | 0.1 | 0.41(0.11-0.76) | 3.14 | 5.22 | 20.36(5.25-37.72) | 3.04 | Middle SDI |
| Guam | 0.09 | 0.26(0.07-0.48) | 4.12 | 4.64 | 12.85(3.46-23.59) | 3.96 | High-middle SDI |
| Guatemala | 4.61 | 0.11(0.03-0.2) | 3.24 | 232.63 | 5.29(1.35-9.87) | 3.19 | Low-middle SDI |
| Guinea | 0.89 | 0.03(0.01-0.06) | 2.23 | 45.32 | 1.37(0.26-2.95) | 2.35 | Low SDI |
| Guinea-Bissau | 0.18 | 0.03(0.01-0.07) | 2.95 | 9.01 | 1.72(0.28-3.67) | 3.03 | Low SDI |
| Guyana | 0.67 | 0.33(0.08-0.64) | 3.62 | 33.79 | 16.63(4.17-31.92) | 3.59 | Middle SDI |
| Haiti | 4.21 | 0.12(0.02-0.29) | 4.11 | 210.94 | 5.96(0.78-14.3) | 4.12 | Low SDI |
| Honduras | 5.42 | 0.19(0.04-0.44) | 3.65 | 272.41 | 9.6(2.02-22.46) | 3.69 | Low-middle SDI |
| Hungary | 6.37 | 0.3(0.08-0.56) | -0.21 | 304.65 | 14.24(3.59-26.73) | -0.26 | High-middle SDI |
| Iceland | 0.12 | 0.15(0.04-0.28) | -0.14 | 6.07 | 7.62(1.89-13.78) | -0.11 | High SDI |
| India | 162 | 0.04(0.01-0.08) | 4.78 | 7905.88 | 2.09(0.28-3.97) | 4.86 | Low-middle SDI |
| Indonesia | 81.4 | 0.11(0.02-0.25) | 6.51 | 4095.31 | 5.44(0.92-12.16) | 6.58 | Low-middle SDI |
| Iran (Islamic Republic of) | 29.5 | 0.13(0.04-0.24) | 4.69 | 1498.19 | 6.45(1.88-11.99) | 4.64 | Middle SDI |
| Iraq | 15.8 | 0.15(0.04-0.31) | 2.37 | 795.17 | 7.54(1.91-15.35) | 2.36 | High-middle SDI |
| Ireland | 1.74 | 0.15(0.04-0.27) | -0.4 | 85.41 | 7.32(1.76-13.13) | -0.37 | Middle SDI |
| Israel | 1.84 | 0.08(0.02-0.16) | -0.95 | 90.1 | 4.05(0.93-7.69) | -0.92 | High-middle SDI |
| Italy | 14.5 | 0.12(0.02-0.24) | 1.37 | 697.53 | 5.75(1.18-11.17) | 1.4 | High SDI |
| Jamaica | 2.23 | 0.29(0.09-0.56) | 2.95 | 112.06 | 14.51(4.26-27.81) | 2.92 | High-middle SDI |
| Japan | 13.1 | 0.05(0-0.12) | 1.31 | 627.16 | 2.52(0.13-5.47) | 1.37 | High SDI |
| Jordan | 4.61 | 0.15(0.04-0.29) | 2.88 | 228.73 | 7.38(2.14-14.23) | 2.88 | Middle SDI |
| Kazakhstan | 11.4 | 0.24(0.06-0.45) | 2.2 | 552.5 | 11.67(2.68-21.71) | 2.22 | High-middle SDI |
| Kenya | 11.6 | 0.09(0.02-0.18) | 5.02 | 589.32 | 4.44(1.02-8.99) | 5.1 | High SDI |
| Kiribati | 0.01 | 0.06(0.02-0.12) | 2.93 | 0.99 | 3.08(0.85-6.18) | 2.94 | High-middle SDI |
| Kuwait | 2.61 | 0.18(0.05-0.31) | 2.95 | 131.38 | 8.99(2.66-15.44) | 2.97 | High SDI |
| Kyrgyzstan | 3.81 | 0.22(0.05-0.42) | 4.91 | 186.63 | 10.82(2.26-20.65) | 4.95 | High-middle SDI |
| Lao People's Democratic Republic | 1.45 | 0.07(0.01-0.16) | 6.53 | 72.72 | 3.66(0.32-7.94) | 6.66 | Low-middle SDI |
| Latvia | 1.54 | 0.4(0.09-0.73) | 1.42 | 72.2 | 18.53(4.33-33.93) | 1.43 | Low-middle SDI |
| Lebanon | 2.56 | 0.17(0.05-0.32) | 2.33 | 128.32 | 8.65(2.29-16.26) | 2.42 | Low-middle SDI |
| Lesotho | 0.7 | 0.14(0.03-0.32) | 3.03 | 35.6 | 7.02(1.35-15.95) | 3.1 | High SDI |
| Liberia | 0.66 | 0.05(0.01-0.1) | 3.86 | 33.05 | 2.37(0.48-4.89) | 3.91 | Low-middle SDI |
| Libya | 6.44 | 0.32(0.08-0.63) | 5.26 | 314.87 | 15.83(3.99-30.65) | 5.24 | Low-middle SDI |
| Lithuania | 1.69 | 0.3(0.06-0.57) | 1.71 | 79.75 | 13.92(2.85-27.09) | 1.73 | High SDI |
| Luxembourg | 0.18 | 0.12(0.03-0.22) | -0.6 | 8.96 | 5.75(1.34-10.75) | -0.57 | High-middle SDI |
| Madagascar | 2.07 | 0.03(0-0.07) | 4.61 | 102.69 | 1.42(0.07-3.37) | 4.68 | Low-middle SDI |
| Malawi | 2.25 | 0.05(0.01-0.1) | 5.08 | 115.46 | 2.3(0.34-5.13) | 5.09 | Low SDI |
| Malaysia | 10.2 | 0.12(0.03-0.25) | 3.68 | 521.92 | 6.19(1.36-12.82) | 3.74 | High-middle SDI |
| Maldives | 0.17 | 0.15(0.03-0.33) | 5.37 | 9.13 | 7.93(1.68-17.51) | 5.49 | High SDI |
| Mali | 0.65 | 0.01(0-0.03) | 3.08 | 32.67 | 0.6(0.1-1.3) | 3.18 | High SDI |
| Malta | 0.19 | 0.21(0.05-0.4) | 2.31 | 9.59 | 10.17(2.56-19.64) | 2.34 | Low SDI |
| Marshall Islands | 0.03 | 0.22(0.05-0.52) | 3.92 | 1.66 | 11.21(2.51-26.03) | 3.83 | Low SDI |
| Mauritania | 0.57 | 0.05(0.01-0.11) | 3.06 | 29.04 | 2.7(0.63-5.67) | 3.11 | High-middle SDI |
| Mauritius | 0.96 | 0.3(0.07-0.56) | 5.08 | 48.81 | 15.42(3.56-28.65) | 5.06 | Middle SDI |
| Mexico | 121 | 0.35(0.1-0.63) | 3.79 | 6032.39 | 17.21(5.03-31.04) | 3.71 | Low SDI |
| Micronesia (Federated States of) | 0.05 | 0.22(0.05-0.49) | 3.35 | 2.95 | 11.27(2.72-25.05) | 3.3 | High-middle SDI |
| Monaco | 0.01 | 0.2(0.04-0.45) | -0.06 | 0.69 | 9.66(2.14-22.28) | -0.02 | Low-middle SDI |
| Mongolia | 1.39 | 0.17(0.03-0.33) | 2.96 | 68.1 | 8.07(1.61-16.21) | 2.97 | Low-middle SDI |
| Montenegro | 0.3 | 0.21(0.05-0.4) | 1.63 | 14.77 | 10.26(2.37-18.93) | 1.65 | High-middle SDI |
| Morocco | 12 | 0.12(0.03-0.26) | 4.23 | 588.19 | 6.07(1.36-12.37) | 4.24 | Middle SDI |
| Mozambique | 5.07 | 0.07(0.01-0.15) | 4.56 | 252.57 | 3.33(0.46-7.54) | 4.67 | Low-middle SDI |
| Myanmar | 12.9 | 0.09(0.01-0.2) | 3.72 | 644.5 | 4.26(0.53-9.55) | 3.74 | High SDI |
| Namibia | 0.59 | 0.09(0.02-0.19) | 3.53 | 29.68 | 4.49(1.06-9.56) | 3.55 | Low-middle SDI |
| Nauru | 0 | 0.24(0.06-0.58) | 2.12 | 0.35 | 12.29(2.87-29.89) | 2.12 | High-middle SDI |
| Nepal | 3.73 | 0.04(0.01-0.1) | 5.64 | 186.29 | 2.05(0.24-4.9) | 5.71 | Low-middle SDI |
| Netherlands | 4.37 | 0.12(0.03-0.22) | 0.05 | 216.61 | 5.9(1.29-11.13) | 0.12 | Low SDI |
| New Zealand | 1.5 | 0.13(0.03-0.22) | -0.69 | 74.83 | 6.26(1.54-11.13) | -0.69 | Low-middle SDI |
| Nicaragua | 2.21 | 0.12(0.03-0.24) | 3.33 | 111.71 | 6.16(1.59-11.92) | 3.27 | Low-middle SDI |
| Niger | 0.59 | 0.01(0-0.03) | 1.1 | 29.06 | 0.55(0.06-1.26) | 1.12 | Middle SDI |
| Nigeria | 20.4 | 0.04(0.01-0.07) | 4.2 | 1025.22 | 1.79(0.36-3.56) | 4.27 | Low SDI |
| Niue | 0 | 0.28(0.08-0.55) | 3.2 | 0.06 | 14.75(4.1-28.55) | 3.33 | High SDI |
| North Macedonia | 1.38 | 0.26(0.06-0.51) | 1.81 | 66.45 | 12.52(2.9-24.53) | 1.82 | High SDI |
| Northern Mariana Islands | 0.04 | 0.35(0.1-0.62) | 4.87 | 1.99 | 17.36(4.66-30.45) | 4.69 | Low-middle SDI |
| Norway | 1.22 | 0.1(0.02-0.18) | -0.94 | 60.26 | 4.98(1.17-9.05) | -0.9 | Low SDI |
| Oman | 0.63 | 0.06(0.02-0.12) | 3.15 | 32.06 | 3.16(0.92-6.04) | 3.2 | Low SDI |
| Pakistan | 83.8 | 0.14(0.03-0.29) | 5.31 | 4220.17 | 6.97(1.35-14.6) | 5.45 | High-middle SDI |
| Palau | 0 | 0.06(0.01-0.11) | 2.9 | 0.11 | 2.84(0.75-5.46) | 2.74 | High-middle SDI |
| Palestine | 1.67 | 0.13(0.03-0.23) | 2.52 | 85.08 | 6.53(1.74-11.64) | 2.58 | High-middle SDI |
| Panama | 1.74 | 0.16(0.04-0.29) | 6.19 | 88.72 | 8.3(2.05-14.8) | 6.27 | High SDI |
| Papua New Guinea | 1.14 | 0.04(0.01-0.1) | 3.59 | 57.31 | 2.19(0.36-5.03) | 3.65 | High-middle SDI |
| Paraguay | 1.95 | 0.1(0.02-0.21) | 2.88 | 98.58 | 5.2(1.17-10.36) | 2.92 | Low-middle SDI |
| Peru | 17.3 | 0.18(0.04-0.36) | 3.39 | 873.92 | 9.09(2.13-18.29) | 3.42 | High-middle SDI |
| Philippines | 40.2 | 0.14(0.02-0.27) | 4.24 | 2055.97 | 7.01(1.2-13.75) | 4.27 | Middle SDI |
| Poland | 21.5 | 0.24(0.05-0.46) | 0.32 | 1033.12 | 11.7(2.61-22.3) | 0.35 | Middle SDI |
| Portugal | 2.75 | 0.12(0.02-0.22) | 0.72 | 131.45 | 5.65(1.15-10.44) | 0.7 | Low SDI |
| Puerto Rico | 1.86 | 0.25(0.07-0.44) | 4.12 | 92.24 | 12.3(3.34-21.88) | 4.07 | Middle SDI |
| Qatar | 1.17 | 0.21(0.06-0.46) | 1.5 | 60.61 | 10.92(3.15-23.69) | 1.53 | Middle SDI |
| Republic of Korea | 4.78 | 0.04(0-0.1) | 7.32 | 226.94 | 1.96(-0.02-4.52) | 7.22 | Low-middle SDI |
| Republic of Moldova | 1.9 | 0.22(0.05-0.39) | 0.6 | 89.71 | 10.17(2.55-18.05) | 0.6 | High-middle SDI |
| Romania | 10.9 | 0.27(0.06-0.49) | 1.08 | 523.28 | 12.89(2.9-23.32) | 1.07 | High-middle SDI |
| Russian Federation | 115 | 0.34(0.09-0.6) | 1.03 | 5553.82 | 16.39(4.1-29.09) | 1 | High SDI |
| Rwanda | 2.17 | 0.06(0.01-0.14) | 4.61 | 112.11 | 3.18(0.3-6.96) | 4.61 | High SDI |
| Saint Kitts and Nevis | 0.03 | 0.23(0.07-0.42) | 1.69 | 1.75 | 11.21(3.24-20.31) | 1.5 | High SDI |
| Saint Lucia | 0.2 | 0.45(0.13-0.78) | 3.17 | 10.06 | 22.02(6.46-38.49) | 3.09 | High-middle SDI |
| Saint Vincent and the Grenadines | 0.08 | 0.32(0.08-0.62) | 4.56 | 4.41 | 15.88(3.71-30.26) | 4.51 | High-middle SDI |
| Samoa | 0.1 | 0.21(0.06-0.43) | 2.37 | 5.25 | 10.78(3.02-21.86) | 2.38 | High-middle SDI |
| San Marino | 0 | 0.08(0.02-0.18) | 0.73 | 0.28 | 3.94(0.75-8.59) | 0.69 | Low SDI |
| Sao Tome and Principe | 0.05 | 0.1(0.02-0.22) | 3.87 | 2.78 | 4.95(1.08-11.42) | 3.99 | High-middle SDI |
| Saudi Arabia | 19.8 | 0.2(0.05-0.4) | 4.45 | 988.5 | 9.74(2.61-19.8) | 4.41 | Middle SDI |
| Senegal | 1.6 | 0.04(0.01-0.08) | 2.61 | 79.74 | 2.04(0.42-4.23) | 2.63 | Middle SDI |
| Serbia | 5.83 | 0.29(0.07-0.55) | 1.66 | 278.17 | 13.68(3.15-25.97) | 1.7 | Low-middle SDI |
| Seychelles | 0.12 | 0.53(0.14-0.99) | 3.8 | 6.62 | 27.3(7.18-50.53) | 3.8 | High SDI |
| Sierra Leone | 0.48 | 0.02(0-0.05) | 4.01 | 24.17 | 1.06(0.17-2.32) | 4.13 | Low-middle SDI |
| Singapore | 1.51 | 0.1(0.02-0.2) | 5.06 | 72.96 | 5.02(1.14-9.59) | 5.07 | High SDI |
| Slovakia | 3.07 | 0.24(0.06-0.48) | 0.46 | 147.34 | 11.67(2.66-22.67) | 0.51 | Low SDI |
| Slovenia | 0.71 | 0.17(0.04-0.32) | -0.22 | 34.42 | 8.18(1.96-15.3) | -0.16 | High-middle SDI |
| Solomon Islands | 0.17 | 0.1(0.02-0.24) | 4.47 | 8.98 | 5.24(1.09-11.8) | 4.49 | High-middle SDI |
| Somalia | 2.85 | 0.06(0.01-0.13) | 2.24 | 142.48 | 2.95(0.45-6.55) | 2.31 | Low SDI |
| South Africa | 32.4 | 0.21(0.05-0.38) | 2.53 | 1619.98 | 10.46(2.68-18.82) | 2.43 | High SDI |
| South Sudan | 0.36 | 0.02(0-0.04) | 3.07 | 17.56 | 0.76(-0.19-2.03) | 3.06 | High-middle SDI |
| Spain | 14.6 | 0.15(0.03-0.27) | 1 | 709.73 | 7.17(1.67-13.02) | 0.96 | High SDI |
| Sri Lanka | 3.95 | 0.07(0.01-0.15) | 3.61 | 200.37 | 3.55(0.67-7.58) | 3.6 | Low SDI |
| Sudan | 8.92 | 0.08(0.02-0.18) | 3.29 | 456.09 | 4.04(0.84-9.02) | 3.38 | Low SDI |
| Suriname | 0.32 | 0.22(0.05-0.44) | 3.56 | 16.15 | 11.13(2.28-22.25) | 3.63 | High-middle SDI |
| Sweden | 2.19 | 0.1(0.02-0.19) | -1.8 | 108.63 | 4.94(1.15-9.45) | -1.7 | Low SDI |
| Switzerland | 0.98 | 0.05(0.01-0.1) | -0.81 | 48.28 | 2.47(0.48-4.75) | -0.78 | High-middle SDI |
| Syrian Arab Republic | 4.76 | 0.12(0.03-0.23) | 3.86 | 234.27 | 6.02(1.61-11.53) | 3.82 | Middle SDI |
| Taiwan (Province of China) | 8.15 | 0.15(0.03-0.27) | 8.96 | 409.66 | 7.29(1.5-13.44) | 8.99 | Low-middle SDI |
| Tajikistan | 1.46 | 0.06(0.01-0.12) | 2.55 | 72.53 | 2.85(0.54-6.05) | 2.57 | Middle SDI |
| Thailand | 35.3 | 0.22(0.04-0.44) | 6.38 | 1776.92 | 10.9(2.24-21.93) | 6.42 | High SDI |
| Timor-Leste | 0.08 | 0.02(0-0.05) | 9.48 | 4.27 | 1.23(0-2.83) | 9.45 | High SDI |
| Togo | 0.92 | 0.04(0.01-0.09) | 3.86 | 46.62 | 2.16(0.43-4.66) | 3.91 | Middle SDI |
| Tokelau | 0 | 0.23(0.06-0.46) | 3.41 | 0.04 | 12.19(3.19-23.8) | 3.52 | High SDI |
| Tonga | 0.04 | 0.16(0.05-0.31) | 2.5 | 2.08 | 8.21(2.38-15.62) | 2.55 | Low-middle SDI |
| Trinidad and Tobago | 1.54 | 0.45(0.13-0.9) | 2.38 | 76.12 | 22.4(6.24-44.23) | 2.32 | Middle SDI |
| Tunisia | 4.61 | 0.15(0.04-0.29) | 4.43 | 228.2 | 7.44(1.96-14.44) | 4.45 | Low SDI |
| Turkey | 49.9 | 0.23(0.06-0.43) | 1.8 | 2456.35 | 11.34(2.83-21.07) | 1.77 | Low SDI |
| Turkmenistan | 2.03 | 0.16(0.03-0.32) | 4.96 | 98.91 | 7.87(1.47-15.48) | 4.92 | Middle SDI |
| Tuvalu | 0 | 0.16(0.04-0.33) | 2.9 | 0.24 | 8.4(2.14-17.1) | 2.96 | Middle SDI |
| Uganda | 7.77 | 0.07(0.01-0.17) | 5.42 | 397.85 | 3.82(0.58-8.4) | 5.54 | High-middle SDI |
| Ukraine | 35.3 | 0.35(0.07-0.76) | 1.69 | 1694.95 | 16.77(3.54-36.52) | 1.7 | Middle SDI |
| United Arab Emirates | 8.89 | 0.52(0.14-1) | 4.21 | 447.51 | 25.96(7.28-50.08) | 4.12 | High-middle SDI |
| United Kingdom | 23.8 | 0.16(0.04-0.28) | -0.94 | 1189.4 | 7.75(1.82-14.11) | -0.85 | Middle SDI |
| United Republic of Tanzania | 16.3 | 0.11(0.02-0.23) | 4.36 | 828.14 | 5.57(1.2-11.75) | 4.47 | Low-middle SDI |
| United States of America | 138 | 0.18(0.05-0.31) | -0.34 | 6905.69 | 9.12(2.49-15.65) | -0.32 | Low SDI |
| United States Virgin Islands | 0.07 | 0.45(0.12-0.92) | 1.42 | 3.92 | 22.76(5.91-46.82) | 1.51 | High-middle SDI |
| Uruguay | 1.7 | 0.21(0.05-0.37) | 1.22 | 84.58 | 10.16(2.37-18.4) | 1.3 | High SDI |
| Uzbekistan | 11.1 | 0.13(0.03-0.26) | 4.18 | 551.26 | 6.19(1.28-12.54) | 4.21 | High SDI |
| Vanuatu | 0.04 | 0.06(0.01-0.16) | 3.86 | 2.44 | 3.11(0.64-8.02) | 3.89 | Low SDI |
| Venezuela (Bolivarian Republic of) | 18.9 | 0.28(0.08-0.53) | 6.52 | 942.15 | 13.73(3.79-26.28) | 6.34 | High SDI |
| Vietnam | 3.92 | 0.02(0-0.04) | 15.72 | 187.74 | 0.74(-0.02-1.8) | 14.14 | High SDI |
| Yemen | 3.01 | 0.04(0.01-0.08) | 3.97 | 149.19 | 1.79(0.3-3.85) | 4.07 | High-middle SDI |
| Zambia | 4.59 | 0.09(0.01-0.21) | 5.48 | 236.01 | 4.8(0.75-10.55) | 5.52 | Middle SDI |
| Zimbabwe | 7.52 | 0.19(0.04-0.4) | 5.43 | 377.22 | 9.29(1.88-19.45) | 5.47 | Low-middle SDI |

**Table S4 The age-standardized rate of deaths, and DALYs of UC attributable to high BMI in women of reproductive age in 204 countries and territories in 2021.**

| Country or territories | Deaths | |  | DALYs | |  |  |
| --- | --- | --- | --- | --- | --- | --- | --- |
|  | Number | ASR (95% UI) | EAPC | Number | ASR (95% UI) | EAPC | SDI Quintile |
| Afghanistan | 11.4 | 0.16(0.06-0.31) | 1.04 | 564.59 | 7.85(3.19-15.37) | 1.13 | Low SDI |
| Albania | 0.75 | 0.12(0.06-0.23) | 1.16 | 39.03 | 6.36(3.22-11.88) | 1.24 | Middle SDI |
| Algeria | 4.36 | 0.04(0.02-0.06) | 2.21 | 229.52 | 2.04(1.22-3.17) | 2.33 | Middle SDI |
| American Samoa | 0.09 | 0.8(0.34-1.54) | 3.29 | 4.65 | 39.63(17.05-76.14) | 3.21 | High-middle SDI |
| Andorra | 0.01 | 0.07(0.04-0.12) | 1.03 | 0.78 | 3.9(2.04-6.56) | 1.12 | High SDI |
| Angola | 4.06 | 0.05(0.02-0.1) | 1.73 | 199.7 | 2.6(1.23-4.73) | 1.78 | Low-middle SDI |
| Antigua and Barbuda | 0.1 | 0.4(0.29-0.53) | 2.43 | 4.89 | 20.22(14.32-26.42) | 2.31 | High-middle SDI |
| Argentina | 13.51 | 0.11(0.08-0.16) | -1.63 | 702.13 | 5.91(4.12-8.08) | -1.52 | High-middle SDI |
| Armenia | 1.8 | 0.24(0.16-0.34) | -0.26 | 91.03 | 12.35(8.26-16.99) | -0.28 | Middle SDI |
| Australia | 6.17 | 0.1(0.07-0.14) | 1.73 | 343.84 | 5.71(4.03-7.75) | 1.87 | High SDI |
| Austria | 1.54 | 0.08(0.05-0.11) | -0.61 | 87.43 | 4.43(2.94-6.13) | -0.34 | High SDI |
| Azerbaijan | 5.37 | 0.2(0.12-0.33) | 0.25 | 271.39 | 9.9(5.94-16.85) | 0.24 | Middle SDI |
| Bahamas | 0.83 | 0.77(0.5-1.09) | 2.49 | 42 | 38.95(25.3-55.66) | 2.43 | High-middle SDI |
| Bahrain | 0.53 | 0.16(0.09-0.27) | 2.66 | 28.66 | 8.79(4.96-14.26) | 2.78 | High-middle SDI |
| Bangladesh | 12.57 | 0.03(0.01-0.07) | 3.49 | 642.07 | 1.4(0.62-3.37) | 3.51 | Low-middle SDI |
| Barbados | 0.51 | 0.72(0.48-1.05) | 2.19 | 25.86 | 36.33(24.18-52.63) | 2.12 | High-middle SDI |
| Belarus | 6.05 | 0.29(0.18-0.42) | 2.4 | 325.88 | 15.37(9.9-22.82) | 2.61 | High-middle SDI |
| Belgium | 2.53 | 0.1(0.07-0.14) | 0.56 | 139.35 | 5.62(3.8-7.78) | 0.7 | High SDI |
| Belize | 0.59 | 0.49(0.34-0.63) | 2.39 | 29.84 | 24.7(17.45-32.07) | 2.36 | Low-middle SDI |
| Benin | 1.59 | 0.05(0.03-0.08) | 0.42 | 78.89 | 2.43(1.29-4.18) | 0.49 | Low SDI |
| Bermuda | 0.05 | 0.37(0.25-0.52) | 1.16 | 2.64 | 19.6(13.25-27.66) | 1.22 | High SDI |
| Bhutan | 0.09 | 0.04(0.02-0.11) | 1.33 | 4.68 | 2.26(0.94-5.66) | 1.39 | Low-middle SDI |
| Bolivia (Plurinational State of) | 8.49 | 0.27(0.14-0.45) | 0.97 | 418.18 | 13.41(7.13-21.78) | 1 | Low-middle SDI |
| Bosnia and Herzegovina | 1.15 | 0.16(0.09-0.24) | 1.49 | 58.89 | 8.05(4.46-12.46) | 1.61 | High-middle SDI |
| Botswana | 0.66 | 0.1(0.05-0.19) | 2.2 | 32.75 | 4.81(2.31-9.29) | 2.28 | Middle SDI |
| Brazil | 84.52 | 0.14(0.1-0.19) | 1.72 | 4240.06 | 7.22(5.11-9.5) | 1.8 | High-middle SDI |
| Brunei Darussalam | 0.28 | 0.22(0.13-0.36) | 2.94 | 13.73 | 10.99(6.36-17.68) | 2.91 | High-middle SDI |
| Bulgaria | 9.45 | 0.66(0.43-0.94) | 1.17 | 481.54 | 33.81(21.81-48.51) | 1.24 | High-middle SDI |
| Burkina Faso | 1.33 | 0.02(0.01-0.04) | 0.11 | 65.92 | 1.2(0.63-2.12) | 0.14 | Low SDI |
| Burundi | 0.7 | 0.02(0.01-0.04) | -0.11 | 35.91 | 1.15(0.55-2.12) | -0.05 | Low SDI |
| Cabo Verde | 0.09 | 0.06(0.03-0.14) | 1.59 | 4.48 | 2.98(1.54-7.03) | 1.24 | Low-middle SDI |
| Cambodia | 4.04 | 0.09(0.04-0.17) | 1.26 | 201.47 | 4.46(2.01-8.22) | 2.12 | Low-middle SDI |
| Cameroon | 5.58 | 0.07(0.04-0.13) | 2.11 | 283.08 | 3.61(1.95-6.37) | 0.26 | Low-middle SDI |
| Canada | 11.19 | 0.13(0.09-0.18) | 0.18 | 609.36 | 7.35(5.13-10.07) | 1.02 | High SDI |
| Central African Republic | 0.87 | 0.06(0.03-0.11) | 0.9 | 42.01 | 3.04(1.46-5.38) | 1.61 | Low SDI |
| Chad | 0.89 | 0.02(0.01-0.04) | 1.59 | 43.54 | 1.13(0.56-2) | 0.84 | Low SDI |
| Chile | 3.93 | 0.08(0.06-0.11) | 0.8 | 206.67 | 4.39(3.16-5.86) | -0.1 | High-middle SDI |
| China | 407.29 | 0.13(0.07-0.2) | -0.21 | 21018.42 | 6.6(3.91-10.28) | 1.84 | High-middle SDI |
| Colombia | 14.77 | 0.11(0.07-0.16) | 1.76 | 757.3 | 5.78(3.73-8.45) | 1.75 | Middle SDI |
| Comoros | 0.21 | 0.11(0.06-0.18) | 1.65 | 10.58 | 5.42(2.94-8.62) | 2.07 | Low-middle SDI |
| Congo | 2.18 | 0.15(0.08-0.27) | 2.06 | 107.3 | 7.49(3.86-13.4) | 2.91 | Low-middle SDI |
| Cook Islands | 0.01 | 0.18(0.11-0.28) | 2.82 | 0.4 | 9.36(5.63-14.77) | 0.5 | High-middle SDI |
| Costa Rica | 2.51 | 0.19(0.13-0.27) | 0.42 | 129.82 | 10.03(6.69-13.72) | 2.91 | Middle SDI |
| Croatia | 3.21 | 0.2(0.13-0.3) | 2.86 | 162.16 | 11.44(7.33-16.89) | 1.21 | High-middle SDI |
| Cuba | 1.79 | 0.65(0.43-0.94) | 0.95 | 102.58 | 32.94(21.87-47.82) | 2.19 | Middle SDI |
| Cyprus | 16.28 | 0.08(0.05-0.14) | 2.25 | 819.77 | 4.5(2.54-8.04) | 0.95 | High SDI |
| Czechia | 0.29 | 0.26(0.16-0.38) | 0.67 | 16.07 | 14(9.02-20.37) | 0.05 | High SDI |
| Côte d'Ivoire | 5.97 | 0.05(0.02-0.08) | -0.19 | 321.21 | 2.44(1.25-4.04) | 1.65 | Low-middle SDI |
| Democratic People's Republic of Korea | 5.1 | 0.08(0.04-0.14) | 1.99 | 259.25 | 3.93(1.94-7.18) | 2.01 | Low-middle SDI |
| Democratic Republic of the Congo | 8.84 | 0.04(0.02-0.08) | 2.54 | 433.12 | 2.03(0.99-3.9) | 2.55 | Low SDI |
| Denmark | 1.17 | 0.09(0.06-0.13) | -0.53 | 64.8 | 5.13(3.37-7.08) | -0.3 | High SDI |
| Djibouti | 0.17 | 0.05(0.02-0.11) | 2.32 | 8.36 | 2.6(1.2-5.33) | 2.33 | Low-middle SDI |
| Dominica | 0.07 | 0.42(0.27-0.67) | 2.31 | 3.49 | 21.22(13.3-33.36) | 2.33 | High-middle SDI |
| Dominican Republic | 13.25 | 0.46(0.24-0.75) | 2.84 | 668.54 | 23.08(12.24-37.93) | 2.84 | Middle SDI |
| Ecuador | 14.04 | 0.3(0.19-0.44) | -0.05 | 705.15 | 14.95(9.5-22.02) | 0 | Middle SDI |
| Egypt | 25.62 | 0.1(0.06-0.16) | 0.43 | 1333.66 | 5.14(2.98-8.16) | 0.56 | Low-middle SDI |
| El Salvador | 3.28 | 0.18(0.12-0.28) | 1.63 | 165.08 | 9.29(5.85-14.01) | 1.71 | Low-middle SDI |
| Equatorial Guinea | 0.33 | 0.09(0.04-0.16) | 1.39 | 16.52 | 4.53(2.02-8.17) | 1.58 | Middle SDI |
| Eritrea | 0.68 | 0.04(0.02-0.08) | 1.2 | 33.34 | 2.01(0.98-3.9) | 1.26 | Low SDI |
| Estonia | 0.52 | 0.19(0.12-0.27) | -0.89 | 28.99 | 10.41(6.45-14.98) | -0.64 | High SDI |
| Eswatini | 0.47 | 0.15(0.06-0.29) | 2.14 | 23.49 | 7.45(2.93-14.18) | 2.18 | Low-middle SDI |
| Ethiopia | 5.05 | 0.02(0.01-0.03) | -1.46 | 255.91 | 0.92(0.5-1.67) | -1.36 | Low SDI |
| Fiji | 1.13 | 0.5(0.28-0.8) | 1.83 | 56.43 | 24.71(14.09-40) | 1.82 | Middle SDI |
| Finland | 1.19 | 0.1(0.07-0.14) | -0.61 | 66.47 | 5.85(4.1-8.03) | -0.4 | High SDI |
| France | 13.12 | 0.09(0.06-0.13) | 1.14 | 727.18 | 5.12(3.47-7.06) | 1.31 | High SDI |
| Gabon | 0.64 | 0.13(0.07-0.22) | 1.58 | 31.47 | 6.43(3.36-10.8) | 1.64 | Middle SDI |
| Gambia | 0.37 | 0.06(0.03-0.1) | 2.28 | 18.64 | 3.03(1.64-5.23) | 2.3 | Low SDI |
| Georgia | 3.56 | 0.45(0.32-0.62) | -0.29 | 180.24 | 22.9(16.09-31.25) | -0.28 | High SDI |
| Germany | 13.77 | 0.08(0.06-0.11) | -0.28 | 765.99 | 4.5(3.08-6.14) | -0.09 | High SDI |
| Ghana | 8.22 | 0.09(0.05-0.17) | 2.3 | 416.59 | 4.56(2.47-8.6) | 2.32 | Low-middle SDI |
| Greece | 3.32 | 0.15(0.11-0.21) | 2.54 | 177.63 | 8.2(5.68-11.14) | 2.55 | High-middle SDI |
| Greenland | 0.01 | 0.06(0.03-0.1) | -0.69 | 0.36 | 2.84(1.56-4.97) | -0.7 | High SDI |
| Grenada | 0.13 | 0.51(0.33-0.71) | 1.99 | 6.58 | 25.7(16.5-36.04) | 1.91 | Middle SDI |
| Guam | 0.12 | 0.33(0.21-0.47) | 2.78 | 6.07 | 16.82(10.66-22.98) | 2.64 | High-middle SDI |
| Guatemala | 9.77 | 0.22(0.15-0.31) | 0.59 | 488.84 | 11.12(7.69-15.36) | 0.61 | Low-middle SDI |
| Guinea | 1.82 | 0.05(0.03-0.1) | 0.82 | 91.82 | 2.77(1.42-5.25) | 0.91 | Low SDI |
| Guinea-Bissau | 0.37 | 0.07(0.04-0.12) | 0.88 | 18.38 | 3.5(1.92-5.82) | 0.91 | Low SDI |
| Guyana | 1.24 | 0.61(0.36-0.93) | 3.41 | 61.87 | 30.45(18.06-46.55) | 3.42 | Middle SDI |
| Haiti | 12.91 | 0.36(0.18-0.64) | 1.92 | 648.12 | 18.31(9.46-31.95) | 1.91 | Low SDI |
| Honduras | 9.66 | 0.34(0.18-0.58) | 1.92 | 474.08 | 16.7(8.83-28.62) | 1.88 | Low-middle SDI |
| Hungary | 6.1 | 0.29(0.18-0.4) | -0.79 | 320.65 | 14.99(9.53-20.93) | -0.65 | High-middle SDI |
| Iceland | 0.07 | 0.09(0.06-0.13) | 0.18 | 4.14 | 5.2(3.52-7.22) | 0.35 | High SDI |
| India | 104.31 | 0.03(0.02-0.04) | 2.12 | 5187.17 | 1.37(0.87-2.05) | 2.17 | Low-middle SDI |
| Indonesia | 131.26 | 0.17(0.08-0.28) | 3.62 | 6547.26 | 8.7(4.25-13.93) | 3.6 | Middle SDI |
| Iran (Islamic Republic of) | 18.2 | 0.08(0.03-0.12) | 2.74 | 993.53 | 4.27(1.67-6.4) | 2.88 | High-middle SDI |
| Iraq | 10.79 | 0.1(0.06-0.17) | 1.46 | 563.49 | 5.34(3.01-9.07) | 1.55 | Middle SDI |
| Ireland | 1.09 | 0.09(0.06-0.13) | 0.94 | 60.76 | 5.21(3.56-7.31) | 1.15 | High SDI |
| Israel | 1.29 | 0.06(0.04-0.08) | 0.13 | 70.3 | 3.16(2.07-4.34) | 0.28 | High-middle SDI |
| Italy | 13.84 | 0.11(0.08-0.16) | 4.28 | 805.07 | 6.64(4.62-9.27) | 4.5 | High SDI |
| Jamaica | 4.36 | 0.56(0.34-0.86) | 3.89 | 221.63 | 28.7(17.32-43.31) | 3.9 | Middle SDI |
| Japan | 27.29 | 0.11(0.08-0.15) | 2.78 | 1420.77 | 5.71(4.03-7.9) | 2.94 | High SDI |
| Jordan | 3.05 | 0.1(0.06-0.16) | 0.66 | 162.83 | 5.25(3.02-8.7) | 0.82 | High-middle SDI |
| Kazakhstan | 12.04 | 0.25(0.17-0.34) | 0.1 | 617.42 | 13.04(8.93-17.68) | 0.2 | High-middle SDI |
| Kenya | 6.91 | 0.05(0.03-0.09) | 3.22 | 348.81 | 2.63(1.44-4.58) | 3.25 | Low-middle SDI |
| Kiribati | 0.17 | 0.52(0.17-0.93) | 1.45 | 8.29 | 25.92(8.52-46.86) | 1.44 | Low-middle SDI |
| Kuwait | 3.71 | 0.25(0.18-0.33) | 5.51 | 209.43 | 14.33(10.24-18.72) | 5.72 | High SDI |
| Kyrgyzstan | 4.16 | 0.24(0.15-0.34) | 0.35 | 212.59 | 12.33(7.72-17.34) | 0.39 | Low-middle SDI |
| Lao People's Democratic Republic | 2.27 | 0.11(0.05-0.2) | 2.02 | 113.49 | 5.72(2.66-9.92) | 2.09 | Low-middle SDI |
| Latvia | 1.3 | 0.33(0.22-0.49) | 0.48 | 69.3 | 17.78(11.45-26.18) | 0.62 | High SDI |
| Lebanon | 1.3 | 0.09(0.05-0.14) | -0.23 | 70.62 | 4.76(2.58-7.9) | 0.01 | High-middle SDI |
| Lesotho | 0.69 | 0.14(0.06-0.26) | 3.22 | 33.91 | 6.69(3.11-12.51) | 3.28 | Low-middle SDI |
| Liberia | 1.05 | 0.08(0.04-0.13) | 2.01 | 52.25 | 3.74(1.92-6.23) | 2.04 | Low SDI |
| Libya | 3.93 | 0.2(0.11-0.32) | 3.82 | 203.23 | 10.22(5.73-16.81) | 3.87 | High-middle SDI |
| Lithuania | 1.79 | 0.31(0.2-0.47) | 1.3 | 92.45 | 16.14(10.02-24.04) | 1.39 | High SDI |
| Luxembourg | 0.16 | 0.1(0.07-0.14) | -0.57 | 8.91 | 5.72(3.97-7.78) | -0.36 | High SDI |
| Madagascar | 2.84 | 0.04(0.02-0.07) | 1.44 | 141.57 | 1.95(0.97-3.47) | 1.44 | Low SDI |
| Malawi | 2.05 | 0.04(0.02-0.07) | 1.79 | 104.18 | 2.08(1.05-3.69) | 1.79 | Low SDI |
| Malaysia | 11.36 | 0.13(0.08-0.2) | 2.22 | 583.28 | 6.92(4.38-10.13) | 2.29 | High-middle SDI |
| Maldives | 0.04 | 0.04(0.02-0.07) | 0.16 | 2.29 | 1.99(1.13-3.58) | 0.37 | Middle SDI |
| Mali | 2.06 | 0.04(0.02-0.06) | 0.89 | 102.98 | 1.89(0.98-3.28) | 0.95 | Low SDI |
| Malta | 0.13 | 0.14(0.1-0.19) | 2.03 | 7.2 | 7.63(5.2-10.69) | 2.17 | High-middle SDI |
| Marshall Islands | 0.11 | 0.76(0.28-1.7) | 3.17 | 5.61 | 37.85(13.81-84.48) | 3.1 | Low-middle SDI |
| Mauritania | 0.74 | 0.07(0.03-0.12) | 0.62 | 37.35 | 3.48(1.77-5.94) | 0.68 | Low-middle SDI |
| Mauritius | 1.45 | 0.46(0.31-0.61) | 2.3 | 74.47 | 23.53(16.08-31.49) | 2.31 | High-middle SDI |
| Mexico | 65.51 | 0.19(0.13-0.26) | 3.26 | 3270.83 | 9.33(6.31-12.88) | 3.25 | Middle SDI |
| Micronesia (Federated States of) | 0.16 | 0.61(0.29-1.13) | 2.1 | 7.93 | 30.31(14.27-55.66) | 2.05 | Low-middle SDI |
| Monaco | 0.01 | 0.08(0.04-0.13) | 1.03 | 0.32 | 4.42(2.42-7.22) | 1.16 | High SDI |
| Mongolia | 1.56 | 0.19(0.1-0.3) | 1.5 | 78.32 | 9.28(4.91-15.21) | 1.53 | Low-middle SDI |
| Montenegro | 0.29 | 0.2(0.12-0.31) | 0.9 | 15.07 | 10.46(6.43-16.34) | 1 | High-middle SDI |
| Morocco | 4.73 | 0.05(0.03-0.08) | 2.71 | 238.67 | 2.46(1.31-4.16) | 2.75 | Low-middle SDI |
| Mozambique | 5.72 | 0.08(0.03-0.15) | 1.84 | 284.94 | 3.75(1.68-7.29) | 1.88 | Low SDI |
| Myanmar | 20.99 | 0.14(0.07-0.24) | 1.05 | 1044.82 | 6.91(3.59-11.99) | 1.02 | Low-middle SDI |
| Namibia | 0.61 | 0.09(0.05-0.16) | 2.08 | 30.16 | 4.56(2.33-7.83) | 2.11 | Low-middle SDI |
| Nauru | 0.02 | 0.7(0.28-1.36) | 1.16 | 1 | 35.23(14.2-68.11) | 1.17 | Middle SDI |
| Nepal | 2.49 | 0.03(0.01-0.07) | 2.05 | 125.48 | 1.38(0.62-3.31) | 2.09 | Low SDI |
| Netherlands | 3.11 | 0.08(0.06-0.12) | 0.5 | 164.62 | 4.49(2.93-6.03) | 0.57 | High SDI |
| New Zealand | 2.01 | 0.17(0.12-0.22) | 1 | 103.2 | 8.64(6.03-11.4) | 1.12 | High SDI |
| Nicaragua | 1.37 | 0.08(0.05-0.12) | 1.23 | 69.35 | 3.83(2.29-6.2) | 1.24 | Low-middle SDI |
| Niger | 1.33 | 0.02(0.01-0.04) | -0.51 | 65.6 | 1.23(0.65-2.21) | -0.49 | Low SDI |
| Nigeria | 13.31 | 0.02(0.01-0.04) | 2.03 | 663.42 | 1.16(0.63-2.04) | 2.07 | Low SDI |
| Niue | 0 | 0.6(0.27-1.09) | 1.9 | 0.12 | 30.59(14.04-56.41) | 2.02 | High-middle SDI |
| North Macedonia | 1.46 | 0.28(0.16-0.43) | 0.68 | 74.69 | 14.07(8.32-22.19) | 0.78 | High-middle SDI |
| Northern Mariana Islands | 0.13 | 1.1(0.66-1.7) | 3.58 | 6.27 | 54.75(32.89-84.16) | 3.44 | High-middle SDI |
| Norway | 0.83 | 0.07(0.05-0.09) | -1.37 | 46.07 | 3.8(2.63-5.11) | -1.15 | High SDI |
| Oman | 0.22 | 0.02(0.01-0.04) | 0.96 | 11.85 | 1.17(0.64-2.11) | 1.2 | High-middle SDI |
| Pakistan | 101.4 | 0.17(0.1-0.26) | 3.09 | 5136.75 | 8.48(4.86-13.34) | 3.18 | Low-middle SDI |
| Palau | 0 | 0.08(0.05-0.13) | 2.06 | 0.16 | 4.16(2.45-6.57) | 1.99 | High-middle SDI |
| Palestine | 2.58 | 0.2(0.12-0.29) | 0.58 | 138.03 | 10.6(6.11-15.66) | 0.74 | Middle SDI |
| Panama | 2.05 | 0.19(0.12-0.28) | 3.51 | 105.22 | 9.84(6.09-14.05) | 3.59 | Middle SDI |
| Papua New Guinea | 4.68 | 0.18(0.07-0.39) | 2.01 | 231.24 | 8.84(3.5-19.05) | 2.01 | Low SDI |
| Paraguay | 2.83 | 0.15(0.09-0.25) | 0.7 | 142.29 | 7.5(4.35-12.47) | 0.79 | Middle SDI |
| Peru | 19.66 | 0.2(0.12-0.33) | 0.62 | 997.48 | 10.37(6.15-16.91) | 0.7 | Middle SDI |
| Philippines | 45.38 | 0.15(0.09-0.23) | 2.38 | 2296.08 | 7.83(4.38-11.78) | 2.34 | Low-middle SDI |
| Poland | 16.89 | 0.19(0.13-0.27) | 0.44 | 879.52 | 9.96(6.59-13.78) | 0.61 | High-middle SDI |
| Portugal | 2.93 | 0.13(0.08-0.17) | -0.23 | 162.2 | 6.97(4.74-9.81) | 0.05 | High-middle SDI |
| Puerto Rico | 2.85 | 0.38(0.26-0.52) | 2.57 | 146.56 | 19.55(13.8-27.04) | 2.56 | High SDI |
| Qatar | 0.64 | 0.12(0.07-0.23) | 0.29 | 36.9 | 6.65(3.85-13.01) | 0.57 | High SDI |
| Republic of Korea | 5.43 | 0.05(0.02-0.08) | 0.05 | 285.18 | 2.46(1.28-4) | 0.22 | High SDI |
| Republic of Moldova | 2.05 | 0.23(0.16-0.31) | 0.45 | 106.34 | 12.06(8.36-16.07) | 0.57 | High-middle SDI |
| Romania | 10.53 | 0.26(0.17-0.37) | 0.28 | 540.47 | 13.31(8.69-19.02) | 0.4 | High-middle SDI |
| Russian Federation | 152.18 | 0.45(0.3-0.6) | 0.47 | 8201.19 | 24.2(16.58-31.96) | 0.6 | High-middle SDI |
| Rwanda | 1.84 | 0.05(0.03-0.1) | 0.11 | 93.76 | 2.66(1.45-4.94) | 0.11 | Low SDI |
| Saint Kitts and Nevis | 0.07 | 0.43(0.28-0.61) | 0.62 | 3.35 | 21.44(13.92-30.38) | 0.4 | High-middle SDI |
| Saint Lucia | 0.26 | 0.57(0.4-0.79) | 2.41 | 13.07 | 28.62(19.72-39.21) | 2.36 | Middle SDI |
| Saint Vincent and the Grenadines | 0.15 | 0.52(0.3-0.79) | 3.53 | 7.25 | 26.07(15.03-39.17) | 3.45 | Middle SDI |
| Samoa | 0.19 | 0.4(0.19-0.72) | 1.89 | 9.76 | 20.07(9.65-36.02) | 1.91 | Low-middle SDI |
| San Marino | 0 | 0.03(0.01-0.05) | 0.98 | 0.1 | 1.47(0.66-2.6) | 1.01 | High SDI |
| Sao Tome and Principe | 0.05 | 0.09(0.05-0.16) | 2.37 | 2.55 | 4.55(2.27-8.15) | 2.43 | Low-middle SDI |
| Saudi Arabia | 12.85 | 0.13(0.07-0.21) | 3.21 | 689.19 | 6.79(3.65-11.27) | 3.37 | High SDI |
| Senegal | 2.52 | 0.06(0.03-0.12) | 0.83 | 124.61 | 3.19(1.68-5.81) | 0.84 | Low SDI |
| Serbia | 4.52 | 0.22(0.12-0.37) | 0.53 | 229.84 | 11.31(6.25-19.11) | 0.63 | High-middle SDI |
| Seychelles | 0.08 | 0.32(0.2-0.45) | 1.77 | 3.97 | 16.38(10.39-23.36) | 1.79 | High-middle SDI |
| Sierra Leone | 0.85 | 0.04(0.02-0.06) | 2.15 | 42.78 | 1.88(1.07-3.14) | 2.22 | Low SDI |
| Singapore | 1.12 | 0.08(0.05-0.11) | 1.72 | 58.59 | 4.03(2.68-5.62) | 1.84 | High SDI |
| Slovakia | 3.67 | 0.29(0.16-0.5) | -0.25 | 188.14 | 14.91(8.14-25.95) | -0.11 | High-middle SDI |
| Slovenia | 0.76 | 0.18(0.12-0.26) | -0.16 | 41.96 | 9.98(6.36-14.5) | 0.08 | High SDI |
| Solomon Islands | 0.69 | 0.4(0.18-0.71) | 2.92 | 33.66 | 19.63(8.85-34.63) | 2.94 | Low SDI |
| Somalia | 3.82 | 0.08(0.04-0.14) | 0.41 | 188.49 | 3.9(1.91-6.9) | 0.44 | Low SDI |
| South Africa | 20.42 | 0.13(0.09-0.2) | 1.25 | 1018.5 | 6.58(4.4-10.18) | 1.21 | High-middle SDI |
| South Sudan | 0.87 | 0.04(0.02-0.07) | 1.36 | 42.73 | 1.84(0.89-3.25) | 1.33 | Low SDI |
| Spain | 11.56 | 0.12(0.08-0.16) | 0.41 | 649.2 | 6.56(4.42-8.98) | 0.62 | High-middle SDI |
| Sri Lanka | 3.64 | 0.06(0.03-0.12) | 0.91 | 190.38 | 3.37(1.56-6.28) | 0.98 | Middle SDI |
| Sudan | 9.33 | 0.08(0.04-0.14) | 1.87 | 485.83 | 4.3(2.04-7.2) | 2 | Low-middle SDI |
| Suriname | 0.36 | 0.25(0.15-0.37) | 3.12 | 17.93 | 12.35(7.3-18.78) | 3.14 | Middle SDI |
| Sweden | 1.11 | 0.05(0.03-0.07) | -0.96 | 62.78 | 2.86(1.89-4.12) | -0.76 | High SDI |
| Switzerland | 1.36 | 0.07(0.04-0.1) | -0.26 | 76.59 | 3.91(2.6-5.59) | -0.11 | High SDI |
| Syrian Arab Republic | 4.68 | 0.12(0.07-0.19) | 1.58 | 244.26 | 6.28(3.84-10.02) | 1.64 | Middle SDI |
| Taiwan (Province of China) | 8.14 | 0.14(0.09-0.2) | 6.19 | 446.96 | 7.96(5.15-10.99) | 6.25 | High SDI |
| Tajikistan | 4.77 | 0.19(0.08-0.4) | 0.45 | 241.97 | 9.52(4.12-20.43) | 0.44 | Low-middle SDI |
| Thailand | 26.1 | 0.16(0.09-0.26) | 4.04 | 1363.96 | 8.37(4.71-13.68) | 4.15 | Middle SDI |
| Timor-Leste | 0.16 | 0.05(0.02-0.08) | 2.47 | 8.28 | 2.39(1.07-4.14) | 2.5 | Low SDI |
| Togo | 1.4 | 0.06(0.03-0.11) | 1.85 | 69.67 | 3.23(1.76-5.44) | 1.86 | Low SDI |
| Tokelau | 0 | 0.57(0.29-0.96) | 1.77 | 0.09 | 29.12(14.96-48.77) | 1.89 | Middle SDI |
| Tonga | 0.1 | 0.38(0.19-0.61) | 1.23 | 4.87 | 19.25(9.49-30.78) | 1.27 | Middle SDI |
| Trinidad and Tobago | 2.47 | 0.73(0.45-1.08) | 2.48 | 124.54 | 36.64(22.55-54.75) | 2.49 | High-middle SDI |
| Tunisia | 2.15 | 0.07(0.04-0.12) | 2.62 | 114.59 | 3.73(2.09-6.26) | 2.75 | Middle SDI |
| Turkey | 36.53 | 0.17(0.1-0.26) | 0.06 | 1942.05 | 8.97(5.31-13.73) | 0.21 | High-middle SDI |
| Turkmenistan | 1.72 | 0.14(0.09-0.2) | 1.01 | 87.09 | 6.93(4.37-10.27) | 0.98 | Middle SDI |
| Tuvalu | 0.01 | 0.48(0.22-0.89) | 0.99 | 0.7 | 24.16(10.88-44.68) | 1.04 | Low-middle SDI |
| Uganda | 8.71 | 0.08(0.05-0.14) | 3.06 | 443.9 | 4.26(2.29-7.14) | 3.13 | Low SDI |
| Ukraine | 45.03 | 0.45(0.22-0.75) | 2.2 | 2265.51 | 22.42(11.37-37.56) | 2.28 | High-middle SDI |
| United Arab Emirates | 5.84 | 0.34(0.2-0.57) | 2.26 | 309.76 | 17.97(10.47-30.55) | 2.34 | High SDI |
| United Kingdom | 17.88 | 0.12(0.08-0.15) | 1.93 | 964.36 | 6.29(4.53-8.26) | 2.07 | High SDI |
| United Republic of Tanzania | 12.48 | 0.08(0.05-0.15) | 1.81 | 626.87 | 4.22(2.3-7.23) | 1.86 | Low SDI |
| United States of America | 172.19 | 0.23(0.17-0.28) | 2.48 | 9803.9 | 12.95(9.49-16.36) | 2.59 | High SDI |
| United States Virgin Islands | 0.07 | 0.39(0.2-0.79) | -0.27 | 3.42 | 19.89(10.22-41.01) | -0.18 | High SDI |
| Uruguay | 1.12 | 0.13(0.09-0.18) | 0.11 | 58.15 | 6.99(4.76-9.63) | 0.22 | High-middle SDI |
| Uzbekistan | 18.42 | 0.21(0.13-0.32) | 1.86 | 936.11 | 10.52(6.42-15.94) | 1.85 | Middle SDI |
| Vanuatu | 0.23 | 0.3(0.14-0.51) | 2.48 | 11.44 | 14.59(7.07-25.02) | 2.48 | Low-middle SDI |
| Venezuela (Bolivarian Republic of) | 18.09 | 0.26(0.16-0.4) | 1.85 | 906.56 | 13.21(7.94-19.72) | 1.87 | Low-middle SDI |
| Vietnam | 5.81 | 0.02(0.01-0.04) | 4.86 | 292.36 | 1.14(0.61-1.91) | 4.83 | Middle SDI |
| Yemen | 3.88 | 0.05(0.02-0.08) | 2.14 | 195.39 | 2.34(1.23-4.13) | 2.23 | Low SDI |
| Zambia | 5.35 | 0.11(0.04-0.27) | 2.62 | 271.96 | 5.53(2.01-13.95) | 2.64 | Low-middle SDI |
| Zimbabwe | 10.47 | 0.26(0.12-0.44) | 4.88 | 519.56 | 12.79(6.13-21.66) | 4.88 | Low-middle SDI |

**Table S5 The age-standardized rate of deaths, and DALYs of OC and UC due to high BMI in women of reproductive age across different age groups in 2021.**

| Age | Sex | OC | | | | UC | | | | |
| --- | --- | --- | --- | --- | --- | --- | --- | --- | --- | --- |
|  |  | Deaths | | DALYs | | Deaths | | DALYs | | |
|  |  | Number | ASR (95% UI) | Number | ASR (95% UI) | Number | ASR (95% UI) | Number | ASR (95% UI) |  |
| 20-24 | Female | 30.6 | 0.01  (0.0014-0.02) | 2181.58 | 0.74  (0.099-1.43) | 20.23 | 0.0069  (0.0047-0.0094) | 1437.42 | 0.49  (0.34-0.67) |  |
| 25-29 | Female | 67.09 | 0.023  (0.0043-0.044) | 4413.08 | 1.52  (0.28-2.88) | 59.07 | 0.02  (0.014-0.027) | 3928.17 | 1.35  (0.92-1.81) |  |
| 30-34 | Female | 138.33 | 0.046  (0.0098-0.085) | 8327.83 | 2.79  (0.59-5.07) | 141.81 | 0.047  (0.032-0.064) | 8811.00 | 2.95  (1.99-3.96) |  |
| 35-39 | Female | 264.2 | 0.095  (0.021-0.17) | 14527.6 | 5.23  (1.18-9.46) | 289.68 | 0.1  (0.07-0.14) | 16778.00 | 6.04  (4.14-8.06) |  |
| 40-44 | Female | 527.28 | 0.21  (0.049-0.38) | 26241.64 | 10.58  (2.46-18.86) | 568.27 | 0.23  (0.16-0.31) | 29835.17 | 12.03  (8.57-16.18) |  |
| 45-49 | Female | 994.56 | 0.42  (0.099-0.75) | 44222.82 | 18.77  (4.42-33.45) | 1122.74 | 0.48  (0.33-0.63) | 53387.66 | 22.66  (15.93-30.00) |  |

**Table S6 SDI Quintile Reference Ranges Used for Stratification**

| Location_name | Lower_bound | Upper_bound |
| --- | --- | --- |
| Low SDI | 0 | 0.46581580319161997 |
| Low-middle SDI | 0.46581580319161997 | 0.6188294452454329 |
| Middle SDI | 0.6188294452454329 | 0.7119746219361235 |
| High-middle SDI | 0.7119746219361235 | 0.8102959891918925 |
| High SDI | 0.8102959891918925 | 1 |

**Table S7 Deaths and DALYs attributable to high BMI for OC among women of reproductive age in 204 countries and territories in 1990 and 2021, with absolute numbers and percentage changes.**

| Country or territories | Deaths | | | DALYs | | |
| --- | --- | --- | --- | --- | --- | --- |
|  | Case-1990 | Case-2021 | Change (%) | Case-1990 | Case-2021 | Change (%) |
| Afghanistan | 0.86 | 5.05 | 485.94 | 41.12 | 249.7 | 507.26 |
| Albania | 0.27 | 0.47 | 74.05 | 13.34 | 22.94 | 71.99 |
| Algeria | 1.45 | 8.69 | 501.48 | 71.16 | 431.54 | 506.47 |
| American Samoa | 0.01 | 0.03 | 191.08 | 0.62 | 1.75 | 182.1 |
| Andorra | 0.01 | 0.01 | 107.55 | 0.25 | 0.52 | 104.83 |
| Angola | 0.16 | 2.89 | 1703.61 | 7.65 | 142.71 | 1765.67 |
| Antigua and Barbuda | 0.02 | 0.06 | 248.51 | 0.96 | 3.22 | 234.9 |
| Argentina | 13.22 | 23.7 | 79.7 | 641.89 | 1174.4 | 82.96 |
| Armenia | 0.81 | 1.15 | 42.94 | 38.91 | 55.28 | 42.09 |
| Australia | 8.09 | 8.11 | 0.24 | 398.73 | 404.38 | 1.42 |
| Austria | 2.48 | 1.64 | -33.95 | 116.94 | 79.91 | -31.67 |
| Azerbaijan | 1.14 | 3.88 | 239.39 | 56.1 | 188.89 | 236.73 |
| Bahamas | 0.18 | 0.59 | 231.96 | 9.1 | 29.47 | 223.83 |
| Bahrain | 0.14 | 1.02 | 643.52 | 7.14 | 51.15 | 616.58 |
| Bangladesh | 0.56 | 22.8 | 3945.95 | 28.44 | 1161.29 | 3983.04 |
| Barbados | 0.14 | 0.27 | 90.56 | 7.24 | 13.41 | 85.08 |
| Belarus | 2.83 | 5.86 | 107.28 | 132.12 | 278.22 | 110.59 |
| Belgium | 3.26 | 3 | -7.92 | 158.31 | 147.86 | -6.6 |
| Belize | 0.03 | 0.17 | 554.86 | 1.34 | 8.63 | 544.09 |
| Benin | 0.19 | 0.97 | 428.35 | 8.99 | 48.65 | 441.06 |
| Bermuda | 0.04 | 0.05 | 24.23 | 2.13 | 2.6 | 22.43 |
| Bhutan | 0.04 | 0.18 | 386.63 | 1.9 | 9.45 | 398.84 |
| Bolivia (Plurinational State of) | 0.6 | 4.83 | 705.01 | 29.63 | 242.5 | 718.37 |
| Bosnia and Herzegovina | 1.12 | 1.41 | 25.63 | 53.35 | 66.94 | 25.48 |
| Botswana | 0.13 | 0.96 | 648.57 | 6.37 | 49.5 | 677.36 |
| Brazil | 28.39 | 101 | 256.49 | 1411.2 | 5073.17 | 259.49 |
| Brunei Darussalam | 0.05 | 0.33 | 623.36 | 2.31 | 16.71 | 622.62 |
| Bulgaria | 5.19 | 5.79 | 11.75 | 247.95 | 277.01 | 11.72 |
| Burkina Faso | 0.11 | 0.53 | 406.28 | 5.15 | 26.23 | 409.03 |
| Burundi | 0.05 | 0.41 | 700.47 | 2.54 | 21.09 | 728.91 |
| Cabo Verde | 0 | 0.06 | 1288.59 | 0.24 | 3.31 | 1302.36 |
| Cambodia | 0.17 | 2.32 | 1245.24 | 8.26 | 115.46 | 1296.98 |
| Cameroon | 0.65 | 4.15 | 541.81 | 31.95 | 212.65 | 565.5 |
| Canada | 10.02 | 11.8 | 18.25 | 491 | 585.46 | 19.24 |
| Central African Republic | 0.06 | 0.44 | 606.93 | 3.04 | 21.85 | 618.42 |
| Chad | 0.06 | 0.3 | 444.68 | 2.68 | 14.77 | 450.56 |
| Chile | 3.4 | 9.04 | 165.84 | 164.85 | 448.12 | 171.84 |
| China | 24.43 | 217 | 788.34 | 1174.44 | 10478.98 | 792.25 |
| Colombia | 6.24 | 26.8 | 330.01 | 307.86 | 1343.76 | 336.48 |
| Comoros | 0.03 | 0.24 | 789.74 | 1.37 | 12.32 | 798.83 |
| Congo | 0.11 | 1.63 | 1335.25 | 5.43 | 81.72 | 1405.16 |
| Cook Islands | 0 | 0 | 87.62 | 0.14 | 0.26 | 86.7 |
| Costa Rica | 0.38 | 2.41 | 533.96 | 19.2 | 121.6 | 533.25 |
| Croatia | 0.76 | 4.28 | 466.44 | 37.35 | 216.05 | 478.44 |
| Cuba | 1.96 | 2.13 | 9.06 | 92.96 | 103.02 | 10.82 |
| Cyprus | 1.83 | 5.73 | 213.35 | 90.6 | 277.08 | 205.82 |
| Czechia | 0.11 | 0.41 | 273.73 | 5.34 | 20.35 | 280.8 |
| Côte d'Ivoire | 5.81 | 4.7 | -18.97 | 275.15 | 226.01 | -17.86 |
| Democratic People's Republic of Korea | 0.03 | 0.48 | 1693.79 | 1.5 | 24.52 | 1534.38 |
| Democratic Republic of the Congo | 0.28 | 5.65 | 1894.43 | 13.64 | 278 | 1937.86 |
| Denmark | 1.66 | 1.11 | -32.75 | 77.81 | 54.49 | -29.97 |
| Djibouti | 0.01 | 0.12 | 1431.22 | 0.4 | 6.22 | 1438.95 |
| Dominica | 0.01 | 0.02 | 129.31 | 0.62 | 1.42 | 130.21 |
| Dominican Republic | 0.63 | 3.09 | 394.44 | 31.31 | 155.51 | 396.68 |
| Ecuador | 0.4 | 9.78 | 2370.18 | 19.97 | 494.39 | 2375.81 |
| Egypt | 5.34 | 34.7 | 552 | 264.82 | 1758.42 | 564 |
| El Salvador | 0.91 | 4.03 | 344.88 | 45.46 | 202.32 | 345.04 |
| Equatorial Guinea | 0.02 | 0.33 | 1538.46 | 0.97 | 16.99 | 1642.7 |
| Eritrea | 0.05 | 0.38 | 615.31 | 2.55 | 18.95 | 641.77 |
| Estonia | 1.01 | 0.57 | -42.73 | 48.27 | 27.87 | -42.26 |
| Eswatini | 0.17 | 0.65 | 295.54 | 8.36 | 33.53 | 301.24 |
| Ethiopia | 1.24 | 5.19 | 320.32 | 59.51 | 257.84 | 333.29 |
| Fiji | 0.15 | 0.31 | 114.58 | 7.33 | 15.65 | 113.49 |
| Finland | 1.77 | 1.27 | -28.21 | 85.93 | 63.56 | -26.03 |
| France | 9.33 | 12.2 | 31.59 | 455.23 | 611.58 | 34.35 |
| Gabon | 0.11 | 0.71 | 529.6 | 5.63 | 35.94 | 538.55 |
| Gambia | 0.03 | 0.2 | 716.89 | 1.23 | 10.3 | 734.75 |
| Georgia | 0.83 | 2.63 | 218.11 | 39.33 | 127.48 | 224.11 |
| Germany | 32.68 | 21.6 | -33.62 | 1573.24 | 1067.83 | -32.13 |
| Ghana | 0.54 | 5.68 | 953.72 | 27.16 | 289.92 | 967.29 |
| Greece | 2.56 | 4.38 | 71.24 | 124.3 | 209.7 | 68.7 |
| Greenland | 0.04 | 0.04 | -6.74 | 2.2 | 1.96 | -11.03 |
| Grenada | 0.03 | 0.1 | 243.12 | 1.56 | 5.22 | 233.32 |
| Guam | 0.03 | 0.09 | 261.69 | 1.35 | 4.64 | 244.54 |
| Guatemala | 0.72 | 4.61 | 542.3 | 36.74 | 232.63 | 533.24 |
| Guinea | 0.18 | 0.89 | 382.07 | 9.07 | 45.32 | 399.53 |
| Guinea-Bissau | 0.03 | 0.18 | 454.9 | 1.59 | 9.01 | 467.32 |
| Guyana | 0.23 | 0.67 | 199.3 | 11.4 | 33.79 | 196.49 |
| Haiti | 0.53 | 4.21 | 701.59 | 26.24 | 210.94 | 703.98 |
| Honduras | 0.66 | 5.42 | 718.57 | 32.91 | 272.41 | 727.85 |
| Hungary | 8.07 | 6.37 | -20.94 | 391.67 | 304.65 | -22.22 |
| Iceland | 0.1 | 0.12 | 17.66 | 5.11 | 6.07 | 18.76 |
| India | 20.38 | 162 | 697.09 | 969.61 | 7905.88 | 715.37 |
| Indonesia | 7.35 | 81.4 | 1008.56 | 361.55 | 4095.31 | 1032.71 |
| Iran (Islamic Republic of) | 3.88 | 29.5 | 661.93 | 199.59 | 1498.19 | 650.63 |
| Iraq | 2.95 | 15.8 | 437.57 | 148.47 | 795.17 | 435.57 |
| Ireland | 1.5 | 1.74 | 16.57 | 72.47 | 85.41 | 17.85 |
| Israel | 1.36 | 1.84 | 35.83 | 65.79 | 90.1 | 36.96 |
| Italy | 11.21 | 14.5 | 29.53 | 534 | 697.53 | 30.62 |
| Jamaica | 0.7 | 2.23 | 219.48 | 35.43 | 112.06 | 216.25 |
| Japan | 11.38 | 13.1 | 15.95 | 531.28 | 627.16 | 18.05 |
| Jordan | 0.51 | 4.61 | 795.33 | 25.51 | 228.73 | 796.8 |
| Kazakhstan | 5.08 | 11.4 | 126.21 | 242.74 | 552.5 | 127.62 |
| Kenya | 0.99 | 11.6 | 1071.49 | 49.26 | 589.32 | 1096.41 |
| Kiribati | 0 | 0.01 | 319.25 | 0.24 | 0.99 | 319.63 |
| Kuwait | 0.3 | 2.61 | 769.24 | 15.02 | 131.38 | 774.72 |
| Kyrgyzstan | 0.52 | 3.81 | 627.49 | 25.35 | 186.63 | 636.06 |
| Lao People's Democratic Republic | 0.1 | 1.45 | 1356.7 | 4.8 | 72.72 | 1415.36 |
| Latvia | 1.66 | 1.54 | -6.93 | 77.35 | 72.2 | -6.66 |
| Lebanon | 0.63 | 2.56 | 304.86 | 30.9 | 128.32 | 315.34 |
| Lesotho | 0.21 | 0.7 | 237.68 | 10.31 | 35.6 | 245.15 |
| Liberia | 0.08 | 0.66 | 714.69 | 4 | 33.05 | 726.42 |
| Libya | 0.6 | 6.44 | 969.86 | 29.62 | 314.87 | 963.01 |
| Lithuania | 1.61 | 1.69 | 5.07 | 75.44 | 79.75 | 5.7 |
| Luxembourg | 0.14 | 0.18 | 32.88 | 6.68 | 8.96 | 34.18 |
| Madagascar | 0.19 | 2.07 | 986.3 | 9.24 | 102.69 | 1011.08 |
| Malawi | 0.22 | 2.25 | 930.54 | 11.18 | 115.46 | 932.75 |
| Malaysia | 1.79 | 10.2 | 476.44 | 88.79 | 521.92 | 487.83 |
| Maldives | 0.01 | 0.17 | 1125.51 | 0.72 | 9.13 | 1167.4 |
| Mali | 0.09 | 0.65 | 627.42 | 4.36 | 32.67 | 649.79 |
| Malta | 0.1 | 0.19 | 101.92 | 4.7 | 9.59 | 103.93 |
| Marshall Islands | 0.01 | 0.03 | 399.07 | 0.34 | 1.66 | 386.72 |
| Mauritania | 0.1 | 0.57 | 481.27 | 4.92 | 29.04 | 490.65 |
| Mauritius | 0.2 | 0.96 | 392.68 | 9.96 | 48.81 | 389.96 |
| Mexico | 23.98 | 121 | 407.46 | 1217.48 | 6032.39 | 395.48 |
| Micronesia (Federated States of) | 0.02 | 0.05 | 213.61 | 0.96 | 2.95 | 208.64 |
| Monaco | 0.01 | 0.01 | 0.4 | 0.68 | 0.69 | 1.9 |
| Mongolia | 0.34 | 1.39 | 307.41 | 16.69 | 68.1 | 308 |
| Montenegro | 0.2 | 0.3 | 52.43 | 9.65 | 14.77 | 53.14 |
| Morocco | 2.17 | 12 | 455.89 | 105.5 | 588.19 | 457.52 |
| Mozambique | 0.53 | 5.07 | 857.56 | 25.49 | 252.57 | 890.99 |
| Myanmar | 2.87 | 12.9 | 350.14 | 142.53 | 644.5 | 352.17 |
| Namibia | 0.1 | 0.59 | 471.22 | 5.15 | 29.68 | 475.89 |
| Nauru | 0 | 0 | 124.36 | 0.16 | 0.35 | 124.46 |
| Nepal | 0.34 | 3.73 | 986.59 | 16.76 | 186.29 | 1011.85 |
| Netherlands | 4.66 | 4.37 | -5.95 | 225.6 | 216.61 | -3.98 |
| New Zealand | 1.41 | 1.5 | 6.75 | 70.09 | 74.83 | 6.77 |
| Nicaragua | 0.4 | 2.21 | 454.09 | 20.48 | 111.71 | 445.39 |
| Niger | 0.14 | 0.59 | 330.11 | 6.71 | 29.06 | 333.43 |
| Nigeria | 2.02 | 20.4 | 914.48 | 99.01 | 1025.22 | 935.43 |
| Niue | 0 | 0 | 105.74 | 0.03 | 0.06 | 114.08 |
| North Macedonia | 0.76 | 1.38 | 81.8 | 36.44 | 66.45 | 82.39 |
| Northern Mariana Islands | 0.01 | 0.04 | 261.99 | 0.58 | 1.99 | 242.76 |
| Norway | 1.43 | 1.22 | -14.27 | 69.44 | 60.26 | -13.22 |
| Oman | 0.08 | 0.63 | 684.36 | 4.03 | 32.06 | 696.09 |
| Pakistan | 6.58 | 83.8 | 1174.22 | 317.97 | 4220.17 | 1227.21 |
| Palau | 0 | 0 | 121.82 | 0.05 | 0.11 | 111.32 |
| Palestine | 0.26 | 1.67 | 537.3 | 13.1 | 85.08 | 549.29 |
| Panama | 0.16 | 1.74 | 1022.9 | 7.71 | 88.72 | 1050.65 |
| Papua New Guinea | 0.14 | 1.14 | 711.19 | 6.95 | 57.31 | 724.4 |
| Paraguay | 0.4 | 1.95 | 386.65 | 20.05 | 98.58 | 391.75 |
| Peru | 3.49 | 17.3 | 397.92 | 174.18 | 873.92 | 401.75 |
| Philippines | 5.89 | 40.2 | 583.73 | 297.85 | 2055.97 | 590.26 |
| Poland | 20.77 | 21.5 | 3.78 | 987.5 | 1033.12 | 4.62 |
| Portugal | 2.4 | 2.75 | 14.89 | 115.18 | 131.45 | 14.13 |
| Puerto Rico | 0.68 | 1.86 | 173.74 | 34.22 | 92.24 | 169.57 |
| Qatar | 0.11 | 1.17 | 1012.83 | 5.4 | 60.61 | 1021.74 |
| Republic of Korea | 0.58 | 4.78 | 718.79 | 28.5 | 226.94 | 696.41 |
| Republic of Moldova | 2.03 | 1.9 | -5.76 | 95.22 | 89.71 | -5.78 |
| Romania | 10.84 | 10.9 | 0.79 | 521.27 | 523.28 | 0.39 |
| Russian Federation | 91.62 | 115 | 25.67 | 4450.54 | 5553.82 | 24.79 |
| Rwanda | 0.25 | 2.17 | 777.68 | 12.77 | 112.11 | 778.24 |
| Saint Kitts and Nevis | 0.01 | 0.03 | 162.99 | 0.71 | 1.75 | 148.03 |
| Saint Lucia | 0.06 | 0.2 | 252.63 | 2.92 | 10.06 | 244.34 |
| Saint Vincent and the Grenadines | 0.02 | 0.08 | 320.71 | 1.07 | 4.41 | 313.65 |
| Samoa | 0.04 | 0.1 | 173.12 | 1.92 | 5.25 | 173.51 |
| San Marino | 0 | 0 | 43.65 | 0.2 | 0.28 | 41.63 |
| Sao Tome and Principe | 0.01 | 0.05 | 609.27 | 0.38 | 2.78 | 635.96 |
| Saudi Arabia | 1.66 | 19.8 | 1099.66 | 83.38 | 988.5 | 1085.59 |
| Senegal | 0.32 | 1.6 | 403.75 | 15.74 | 79.74 | 406.66 |
| Serbia | 4.03 | 5.83 | 44.83 | 189.6 | 278.17 | 46.71 |
| Seychelles | 0.03 | 0.12 | 325.69 | 1.56 | 6.62 | 325.44 |
| Sierra Leone | 0.06 | 0.48 | 665.22 | 3.05 | 24.17 | 693.28 |
| Singapore | 0.21 | 1.51 | 618.99 | 10.1 | 72.96 | 622.05 |
| Slovakia | 2.81 | 3.07 | 9.65 | 132.32 | 147.34 | 11.35 |
| Slovenia | 0.9 | 0.71 | -21.15 | 42.77 | 34.42 | -19.52 |
| Solomon Islands | 0.02 | 0.17 | 793.72 | 1 | 8.98 | 799.04 |
| Somalia | 0.5 | 2.85 | 467.42 | 24.6 | 142.48 | 479.25 |
| South Africa | 9.35 | 32.4 | 247.57 | 479.47 | 1619.98 | 237.87 |
| South Sudan | 0.08 | 0.36 | 357.9 | 3.84 | 17.56 | 356.79 |
| Spain | 10.47 | 14.6 | 39.96 | 513.33 | 709.73 | 38.26 |
| Sri Lanka | 1.07 | 3.95 | 269.53 | 54.4 | 200.37 | 268.32 |
| Sudan | 1.36 | 8.92 | 558.23 | 67.49 | 456.09 | 575.8 |
| Suriname | 0.07 | 0.32 | 340.99 | 3.58 | 16.15 | 351.02 |
| Sweden | 3.61 | 2.19 | -39.05 | 172.62 | 108.63 | -37.07 |
| Switzerland | 1.14 | 0.98 | -13.54 | 55.41 | 48.28 | -12.87 |
| Syrian Arab Republic | 1.04 | 4.76 | 357.06 | 51.98 | 234.27 | 350.73 |
| Taiwan (Province of China) | 0.56 | 8.15 | 1360.01 | 27.8 | 409.66 | 1373.35 |
| Tajikistan | 0.32 | 1.46 | 360.35 | 15.68 | 72.53 | 362.47 |
| Thailand | 5.09 | 35.3 | 593.7 | 253.19 | 1776.92 | 601.81 |
| Timor-Leste | 0 | 0.08 | 2982.13 | 0.14 | 4.27 | 2956.9 |
| Togo | 0.11 | 0.92 | 717.68 | 5.61 | 46.62 | 730.64 |
| Tokelau | 0 | 0 | 157.65 | 0.01 | 0.04 | 166.19 |
| Tonga | 0.02 | 0.04 | 143.32 | 0.84 | 2.08 | 146.72 |
| Trinidad and Tobago | 0.67 | 1.54 | 129.35 | 33.85 | 76.12 | 124.86 |
| Tunisia | 0.8 | 4.61 | 475.66 | 39.43 | 228.2 | 478.69 |
| Turkey | 18.93 | 49.9 | 163.99 | 936.77 | 2456.35 | 162.21 |
| Turkmenistan | 0.32 | 2.03 | 537.46 | 15.71 | 98.91 | 529.69 |
| Tuvalu | 0 | 0 | 189.7 | 0.08 | 0.24 | 194.6 |
| Uganda | 0.56 | 7.77 | 1289.08 | 27.61 | 397.85 | 1341.09 |
| Ukraine | 26.39 | 35.3 | 34.09 | 1258.21 | 1694.95 | 34.71 |
| United Arab Emirates | 0.49 | 8.89 | 1710.7 | 25.44 | 447.51 | 1658.96 |
| United Kingdom | 29.61 | 23.8 | -19.38 | 1435.72 | 1189.4 | -17.16 |
| United Republic of Tanzania | 1.76 | 16.3 | 828.98 | 86.2 | 828.14 | 860.72 |
| United States of America | 136.02 | 138 | 1.77 | 6752.08 | 6905.69 | 2.28 |
| United States Virgin Islands | 0.08 | 0.07 | -7.36 | 4.12 | 3.92 | -4.89 |
| Uruguay | 1.06 | 1.7 | 61.55 | 51.19 | 84.58 | 65.22 |
| Uzbekistan | 1.73 | 11.1 | 547.39 | 84.47 | 551.26 | 552.61 |
| Vanuatu | 0.01 | 0.04 | 623.85 | 0.33 | 2.44 | 629.1 |
| Venezuela (Bolivarian Republic of) | 1.89 | 18.9 | 905.01 | 98.89 | 942.15 | 852.75 |
| Vietnam | 0.03 | 3.92 | 13713.69 | 2.08 | 187.74 | 8928.14 |
| Yemen | 0.3 | 3.01 | 919.49 | 14.18 | 149.19 | 952 |
| Zambia | 0.33 | 4.59 | 1299.33 | 16.68 | 236.01 | 1314.61 |
| Zimbabwe | 0.86 | 7.52 | 770.92 | 42.84 | 377.22 | 780.58 |

**Table S8 Deaths and DALYs attributable to high BMI for UC among women of reproductive age in 204 countries and territories in 1990 and 2021, with absolute numbers and percentage changes.**

| Country or territories | Deaths | | | DALYs | | |
| --- | --- | --- | --- | --- | --- | --- |
|  | Case-1990 | Case-2021 | Change (%) | Case-1990 | Case-2021 | Change (%) |
| Afghanistan | 2.53 | 11.4 | 350.39 | 121.73 | 564.59 | 363.8 |
| Albania | 0.72 | 0.75 | 5.12 | 36.27 | 39.03 | 7.61 |
| Algeria | 1.15 | 4.36 | 280.12 | 58.13 | 229.52 | 294.81 |
| American Samoa | 0.04 | 0.09 | 166.2 | 1.79 | 4.65 | 160 |
| Andorra | 0.01 | 0.01 | 84.07 | 0.41 | 0.78 | 89.24 |
| Angola | 0.71 | 4.06 | 467.96 | 34.65 | 199.7 | 476.35 |
| Antigua and Barbuda | 0.03 | 0.1 | 213.18 | 1.62 | 4.89 | 202.26 |
| Argentina | 15.2 | 13.51 | -11.14 | 762.04 | 702.13 | -7.86 |
| Armenia | 2.3 | 1.8 | -21.57 | 116.69 | 91.03 | -21.99 |
| Australia | 2.68 | 6.17 | 129.8 | 143.54 | 343.84 | 139.55 |
| Austria | 1.87 | 1.54 | -17.53 | 97.53 | 87.43 | -10.36 |
| Azerbaijan | 3.41 | 5.37 | 57.73 | 172.56 | 271.39 | 57.27 |
| Bahamas | 0.26 | 0.83 | 218.28 | 13.42 | 42 | 212.92 |
| Bahrain | 0.08 | 0.53 | 537.58 | 4.33 | 28.66 | 562.05 |
| Bangladesh | 2.32 | 12.57 | 441.14 | 117.88 | 642.07 | 444.67 |
| Barbados | 0.25 | 0.51 | 103.19 | 12.97 | 25.86 | 99.33 |
| Belarus | 3.46 | 6.05 | 74.66 | 175.13 | 325.88 | 86.09 |
| Belgium | 2.09 | 2.53 | 20.98 | 110.42 | 139.35 | 26.2 |
| Belize | 0.1 | 0.59 | 497.16 | 5.05 | 29.84 | 491.42 |
| Benin | 0.47 | 1.59 | 237.63 | 22.91 | 78.89 | 244.41 |
| Bermuda | 0.04 | 0.05 | 12.75 | 2.3 | 2.64 | 14.67 |
| Bhutan | 0.04 | 0.09 | 118.01 | 2.11 | 4.68 | 122.12 |
| Bolivia (Plurinational State of) | 3.09 | 8.49 | 174.89 | 150.65 | 418.18 | 177.58 |
| Bosnia and Herzegovina | 1.16 | 1.15 | -0.56 | 57.15 | 58.89 | 3.03 |
| Botswana | 0.16 | 0.66 | 314.41 | 7.7 | 32.75 | 325.1 |
| Brazil | 33.09 | 84.52 | 155.45 | 1616.35 | 4240.06 | 162.32 |
| Brunei Darussalam | 0.06 | 0.28 | 354.31 | 3.05 | 13.73 | 350.44 |
| Bulgaria | 9.57 | 9.45 | -1.33 | 477.23 | 481.54 | 0.9 |
| Burkina Faso | 0.49 | 1.33 | 171.73 | 24.09 | 65.92 | 173.63 |
| Burundi | 0.29 | 0.7 | 140.26 | 14.7 | 35.91 | 144.25 |
| Cabo Verde | 0.03 | 0.09 | 179.5 | 1.61 | 4.48 | 177.57 |
| Cambodia | 1.17 | 4.04 | 244.46 | 58.21 | 201.47 | 246.11 |
| Cameroon | 1.61 | 5.58 | 247.69 | 79.27 | 283.08 | 257.13 |
| Canada | 7.51 | 11.19 | 48.87 | 395.18 | 609.36 | 54.2 |
| Central African Republic | 0.25 | 0.87 | 251.74 | 11.85 | 42.01 | 254.52 |
| Chad | 0.24 | 0.89 | 272.91 | 11.56 | 43.54 | 276.61 |
| Chile | 3.22 | 3.93 | 22.09 | 163.34 | 206.67 | 26.53 |
| China | 239.79 | 407.29 | 69.85 | 12076.89 | 21018.42 | 74.04 |
| Colombia | 5.9 | 14.77 | 150.23 | 292.96 | 757.3 | 158.49 |
| Comoros | 0.06 | 0.21 | 248.46 | 3.02 | 10.58 | 249.89 |
| Congo | 0.36 | 2.18 | 501.42 | 17.36 | 107.3 | 518.02 |
| Cook Islands | 0.01 | 0.01 | 6.65 | 0.37 | 0.4 | 9.46 |
| Costa Rica | 0.63 | 2.51 | 299.25 | 32.07 | 129.82 | 304.83 |
| Croatia | 1.79 | 1.79 | 0.04 | 94.5 | 102.58 | 8.56 |
| Cuba | 10.05 | 16.28 | 61.99 | 515.44 | 819.77 | 59.04 |
| Cyprus | 0.13 | 0.29 | 122.45 | 6.63 | 16.07 | 142.19 |
| Czechia | 7.11 | 5.97 | -16 | 354.77 | 321.21 | -9.46 |
| Côte d'Ivoire | 0.81 | 3.21 | 297.38 | 40.1 | 162.16 | 304.38 |
| Democratic People's Republic of Korea | 2.38 | 5.1 | 113.95 | 120.41 | 259.25 | 115.3 |
| Democratic Republic of the Congo | 1.62 | 8.84 | 444.03 | 79.49 | 433.12 | 444.89 |
| Denmark | 1.42 | 1.17 | -17.96 | 73.5 | 64.8 | -11.83 |
| Djibouti | 0.03 | 0.17 | 566.09 | 1.25 | 8.36 | 567.95 |
| Dominica | 0.04 | 0.07 | 95.87 | 1.77 | 3.49 | 97.31 |
| Dominican Republic | 3.63 | 13.25 | 265.45 | 182.98 | 668.54 | 265.36 |
| Ecuador | 7.62 | 14.04 | 84.23 | 376.98 | 705.15 | 87.05 |
| Egypt | 11.34 | 25.62 | 125.92 | 566.69 | 1333.66 | 135.34 |
| El Salvador | 1.47 | 3.28 | 123.42 | 71.98 | 165.08 | 129.34 |
| Equatorial Guinea | 0.06 | 0.33 | 465.6 | 2.75 | 16.52 | 500.03 |
| Eritrea | 0.23 | 0.68 | 203.52 | 10.79 | 33.34 | 209 |
| Estonia | 0.94 | 0.52 | -44.65 | 48.41 | 28.99 | -40.12 |
| Eswatini | 0.15 | 0.47 | 211.66 | 7.44 | 23.49 | 215.7 |
| Ethiopia | 3.24 | 5.05 | 55.88 | 159.35 | 255.91 | 60.6 |
| Fiji | 0.55 | 1.13 | 104.86 | 27.62 | 56.43 | 104.34 |
| Finland | 1.59 | 1.19 | -25.58 | 83.62 | 66.47 | -20.51 |
| France | 9.42 | 13.12 | 39.31 | 494.5 | 727.18 | 47.05 |
| Gabon | 0.18 | 0.64 | 259.41 | 8.61 | 31.47 | 265.67 |
| Gambia | 0.07 | 0.37 | 444.42 | 3.4 | 18.64 | 448.85 |
| Georgia | 6.79 | 3.56 | -47.56 | 343.32 | 180.24 | -47.5 |
| Germany | 17.15 | 13.77 | -19.7 | 898.22 | 765.99 | -14.72 |
| Ghana | 1.57 | 8.22 | 424.65 | 78.94 | 416.59 | 427.76 |
| Greece | 1.78 | 3.32 | 87.13 | 94.67 | 177.63 | 87.63 |
| Greenland | 0.01 | 0.01 | -30.32 | 0.52 | 0.36 | -30.48 |
| Grenada | 0.05 | 0.13 | 142.65 | 2.78 | 6.58 | 136.81 |
| Guam | 0.05 | 0.12 | 142.04 | 2.62 | 6.07 | 131.66 |
| Guatemala | 3.41 | 9.77 | 186.78 | 169.34 | 488.84 | 188.68 |
| Guinea | 0.58 | 1.82 | 213.59 | 28.47 | 91.82 | 222.54 |
| Guinea-Bissau | 0.13 | 0.37 | 195.08 | 6.16 | 18.38 | 198.48 |
| Guyana | 0.44 | 1.24 | 181.63 | 21.95 | 61.87 | 181.86 |
| Haiti | 3.11 | 12.91 | 315.17 | 156.48 | 648.12 | 314.2 |
| Honduras | 1.99 | 9.66 | 385.59 | 98.91 | 474.08 | 379.29 |
| Hungary | 9.25 | 6.1 | -34.06 | 465.05 | 320.65 | -31.05 |
| Iceland | 0.06 | 0.07 | 29.67 | 3.03 | 4.14 | 36.84 |
| India | 29.03 | 104.31 | 259.26 | 1423.26 | 5187.17 | 264.46 |
| Indonesia | 27.76 | 131.26 | 372.84 | 1390.86 | 6547.26 | 370.73 |
| Iran (Islamic Republic of) | 4.28 | 18.2 | 324.81 | 223.94 | 993.53 | 343.65 |
| Iraq | 2.65 | 10.79 | 307 | 134.55 | 563.49 | 318.8 |
| Ireland | 0.62 | 1.09 | 76.31 | 32.26 | 60.76 | 88.34 |
| Israel | 0.68 | 1.29 | 89.72 | 35.37 | 70.3 | 98.74 |
| Italy | 4.45 | 13.84 | 211.35 | 242.01 | 805.07 | 232.67 |
| Jamaica | 1.03 | 4.36 | 323.54 | 52.29 | 221.63 | 323.88 |
| Japan | 15.04 | 27.29 | 81.48 | 747.84 | 1420.77 | 89.98 |
| Jordan | 0.67 | 3.05 | 356.01 | 34.04 | 162.83 | 378.29 |
| Kazakhstan | 10.14 | 12.04 | 18.73 | 504.32 | 617.42 | 22.43 |
| Kenya | 1.01 | 6.91 | 585.02 | 50.54 | 348.81 | 590.17 |
| Kiribati | 0.06 | 0.17 | 167.54 | 3.11 | 8.29 | 166.63 |
| Kuwait | 0.2 | 3.71 | 1761.86 | 10.57 | 209.43 | 1880.86 |
| Kyrgyzstan | 2.27 | 4.16 | 83.38 | 114.42 | 212.59 | 85.8 |
| Lao People's Democratic Republic | 0.6 | 2.27 | 281.45 | 29.18 | 113.49 | 288.95 |
| Latvia | 1.87 | 1.3 | -30.21 | 95.29 | 69.3 | -27.27 |
| Lebanon | 0.71 | 1.3 | 84.06 | 35.61 | 70.62 | 98.32 |
| Lesotho | 0.19 | 0.69 | 258.29 | 9.32 | 33.91 | 263.87 |
| Liberia | 0.23 | 1.05 | 365.7 | 11.09 | 52.25 | 371.01 |
| Libya | 0.56 | 3.93 | 598.26 | 28.65 | 203.23 | 609.39 |
| Lithuania | 1.93 | 1.79 | -7.19 | 96.85 | 92.45 | -4.55 |
| Luxembourg | 0.12 | 0.16 | 34.25 | 6.23 | 8.91 | 43.05 |
| Madagascar | 0.68 | 2.84 | 319.48 | 33.76 | 141.57 | 319.32 |
| Malawi | 0.53 | 2.05 | 284.52 | 27.07 | 104.18 | 284.83 |
| Malaysia | 3.06 | 11.36 | 271 | 153.71 | 583.28 | 279.47 |
| Maldives | 0.02 | 0.04 | 154.65 | 0.84 | 2.29 | 171.34 |
| Mali | 0.55 | 2.06 | 273.9 | 27.04 | 102.98 | 280.81 |
| Malta | 0.07 | 0.13 | 85.08 | 3.72 | 7.2 | 93.29 |
| Marshall Islands | 0.03 | 0.11 | 298.89 | 1.43 | 5.61 | 291.28 |
| Mauritania | 0.27 | 0.74 | 176.86 | 13.24 | 37.35 | 182.09 |
| Mauritius | 0.68 | 1.45 | 114.32 | 34.63 | 74.47 | 115.03 |
| Mexico | 15.14 | 65.51 | 332.7 | 758.3 | 3270.83 | 331.34 |
| Micronesia (Federated States of) | 0.07 | 0.16 | 114.55 | 3.74 | 7.93 | 111.65 |
| Monaco | 0 | 0.01 | 40.79 | 0.22 | 0.32 | 46.53 |
| Mongolia | 0.6 | 1.56 | 161.65 | 29.68 | 78.32 | 163.86 |
| Montenegro | 0.23 | 0.29 | 21.68 | 12.01 | 15.07 | 25.43 |
| Morocco | 1.34 | 4.73 | 252.43 | 66.87 | 238.67 | 256.9 |
| Mozambique | 1.35 | 5.72 | 323.18 | 66.56 | 284.94 | 328.11 |
| Myanmar | 10.47 | 20.99 | 100.39 | 526.55 | 1044.82 | 98.43 |
| Namibia | 0.16 | 0.61 | 269.42 | 8.09 | 30.16 | 272.69 |
| Nauru | 0.01 | 0.02 | 67.26 | 0.6 | 1 | 67.64 |
| Nepal | 0.67 | 2.49 | 272.33 | 33.24 | 125.48 | 277.51 |
| Netherlands | 2.88 | 3.11 | 8.04 | 148.86 | 164.62 | 10.59 |
| New Zealand | 1.12 | 2.01 | 79.98 | 55.37 | 103.2 | 86.4 |
| Nicaragua | 0.47 | 1.37 | 193.34 | 23.55 | 69.35 | 194.49 |
| Niger | 0.51 | 1.33 | 161.28 | 24.9 | 65.6 | 163.5 |
| Nigeria | 2.52 | 13.31 | 428.27 | 124.16 | 663.42 | 434.31 |
| Niue | 0 | 0 | 38.7 | 0.08 | 0.12 | 43.95 |
| North Macedonia | 1.14 | 1.46 | 28.49 | 56.25 | 74.69 | 32.79 |
| Northern Mariana Islands | 0.05 | 0.13 | 146.4 | 2.65 | 6.27 | 136.62 |
| Norway | 1.11 | 0.83 | -25.16 | 57.55 | 46.07 | -19.95 |
| Oman | 0.05 | 0.22 | 302.38 | 2.73 | 11.85 | 334.22 |
| Pakistan | 15.4 | 101.4 | 558.26 | 759.71 | 5136.75 | 576.14 |
| Palau | 0 | 0 | 71.77 | 0.09 | 0.16 | 68.13 |
| Palestine | 0.73 | 2.58 | 252.13 | 37.28 | 138.03 | 270.26 |
| Panama | 0.4 | 2.05 | 409.17 | 20.16 | 105.22 | 421.8 |
| Papua New Guinea | 0.93 | 4.68 | 402.77 | 45.92 | 231.24 | 403.59 |
| Paraguay | 1.13 | 2.83 | 150.85 | 55.32 | 142.29 | 157.21 |
| Peru | 9.16 | 19.66 | 114.65 | 453.13 | 997.48 | 120.13 |
| Philippines | 11.59 | 45.38 | 291.49 | 593.87 | 2296.08 | 286.63 |
| Poland | 15.68 | 16.89 | 7.71 | 773.92 | 879.52 | 13.64 |
| Portugal | 3.41 | 2.93 | -14.25 | 173.47 | 162.2 | -6.5 |
| Puerto Rico | 1.66 | 2.85 | 71.82 | 85.51 | 146.56 | 71.39 |
| Qatar | 0.08 | 0.64 | 667.94 | 4.41 | 36.9 | 735.89 |
| Republic of Korea | 5.83 | 5.43 | -6.81 | 290.79 | 285.18 | -1.93 |
| Republic of Moldova | 2.28 | 2.05 | -9.87 | 113.91 | 106.34 | -6.65 |
| Romania | 13.38 | 10.53 | -21.35 | 662.23 | 540.47 | -18.39 |
| Russian Federation | 143.94 | 152.18 | 5.72 | 7452.02 | 8201.19 | 10.05 |
| Rwanda | 0.82 | 1.84 | 124.37 | 41.81 | 93.76 | 124.25 |
| Saint Kitts and Nevis | 0.04 | 0.07 | 89.14 | 1.89 | 3.35 | 77.2 |
| Saint Lucia | 0.09 | 0.26 | 180.41 | 4.73 | 13.07 | 176.25 |
| Saint Vincent and the Grenadines | 0.05 | 0.15 | 208.83 | 2.4 | 7.25 | 201.97 |
| Samoa | 0.08 | 0.19 | 135.53 | 4.12 | 9.76 | 136.86 |
| San Marino | 0 | 0 | 55 | 0.07 | 0.1 | 56.43 |
| Sao Tome and Principe | 0.01 | 0.05 | 352.45 | 0.55 | 2.55 | 360.75 |
| Saudi Arabia | 1.55 | 12.85 | 729.05 | 79.35 | 689.19 | 768.59 |
| Senegal | 0.86 | 2.52 | 192.83 | 42.46 | 124.61 | 193.45 |
| Serbia | 4.41 | 4.52 | 2.55 | 217.37 | 229.84 | 5.74 |
| Seychelles | 0.03 | 0.08 | 130.44 | 1.71 | 3.97 | 132.01 |
| Sierra Leone | 0.19 | 0.85 | 338.13 | 9.58 | 42.78 | 346.75 |
| Singapore | 0.42 | 1.12 | 163.92 | 21.4 | 58.59 | 173.8 |
| Slovakia | 4.17 | 3.67 | -11.94 | 204.32 | 188.14 | -7.92 |
| Slovenia | 0.95 | 0.76 | -19.66 | 48.39 | 41.96 | -13.29 |
| Solomon Islands | 0.12 | 0.69 | 462.52 | 5.95 | 33.66 | 465.66 |
| Somalia | 1.18 | 3.82 | 224.54 | 57.52 | 188.49 | 227.69 |
| South Africa | 8.65 | 20.42 | 136.11 | 437.58 | 1018.5 | 132.76 |
| South Sudan | 0.32 | 0.87 | 172.11 | 15.85 | 42.73 | 169.66 |
| Spain | 9.9 | 11.56 | 16.76 | 521.31 | 649.2 | 24.53 |
| Sri Lanka | 2.23 | 3.64 | 63.14 | 114.4 | 190.38 | 66.41 |
| Sudan | 2.18 | 9.33 | 328.24 | 109.17 | 485.83 | 345.01 |
| Suriname | 0.09 | 0.36 | 286.93 | 4.6 | 17.93 | 289.37 |
| Sweden | 1.39 | 1.11 | -20.57 | 74.29 | 62.78 | -15.49 |
| Switzerland | 1.33 | 1.36 | 2.66 | 71.24 | 76.59 | 7.5 |
| Syrian Arab Republic | 2.04 | 4.68 | 129.33 | 104.46 | 244.26 | 133.82 |
| Taiwan (Province of China) | 1.24 | 8.14 | 557.89 | 66.76 | 446.96 | 569.46 |
| Tajikistan | 1.97 | 4.77 | 142.36 | 100.27 | 241.97 | 141.33 |
| Thailand | 7.5 | 26.1 | 247.91 | 378.76 | 1363.96 | 260.11 |
| Timor-Leste | 0.04 | 0.16 | 296.32 | 2.07 | 8.28 | 299.32 |
| Togo | 0.31 | 1.4 | 345.67 | 15.56 | 69.67 | 347.82 |
| Tokelau | 0 | 0 | 56.68 | 0.06 | 0.09 | 62.64 |
| Tonga | 0.06 | 0.1 | 65.25 | 2.92 | 4.87 | 66.91 |
| Trinidad and Tobago | 1.05 | 2.47 | 135.88 | 52.66 | 124.54 | 136.5 |
| Tunisia | 0.64 | 2.15 | 234.26 | 32.98 | 114.59 | 247.47 |
| Turkey | 23.61 | 36.53 | 54.67 | 1196.66 | 1942.05 | 62.29 |
| Turkmenistan | 0.89 | 1.72 | 93.98 | 45.23 | 87.09 | 92.54 |
| Tuvalu | 0.01 | 0.01 | 61.94 | 0.43 | 0.7 | 64.65 |
| Uganda | 1.26 | 8.71 | 589.44 | 63.03 | 443.9 | 604.23 |
| Ukraine | 28.71 | 45.03 | 56.89 | 1411.99 | 2265.51 | 60.45 |
| United Arab Emirates | 0.58 | 5.84 | 908.49 | 30.02 | 309.76 | 931.84 |
| United Kingdom | 9.14 | 17.88 | 95.65 | 473.2 | 964.36 | 103.79 |
| United Republic of Tanzania | 2.89 | 12.48 | 331.57 | 142.98 | 626.87 | 338.43 |
| United States of America | 71.3 | 172.19 | 141.51 | 3928.02 | 9803.9 | 149.59 |
| United States Virgin Islands | 0.12 | 0.07 | -44.9 | 6.05 | 3.42 | -43.43 |
| Uruguay | 0.98 | 1.12 | 14.8 | 48.94 | 58.15 | 18.81 |
| Uzbekistan | 5.73 | 18.42 | 221.39 | 292.22 | 936.11 | 220.34 |
| Vanuatu | 0.05 | 0.23 | 378.46 | 2.39 | 11.44 | 378.09 |
| Venezuela (Bolivarian Republic of) | 7.23 | 18.09 | 150.29 | 359.96 | 906.56 | 151.85 |
| Vietnam | 0.89 | 5.81 | 551.04 | 45.25 | 292.36 | 546.15 |
| Yemen | 0.66 | 3.88 | 487.23 | 32.33 | 195.39 | 504.41 |
| Zambia | 0.9 | 5.35 | 495.68 | 45.31 | 271.96 | 500.18 |
| Zimbabwe | 1.42 | 10.47 | 638.91 | 70.2 | 519.56 | 640.13 |
